# Bayesian correlated clustering to integrate multiple datasets

**DOI:** 10.1093/bioinformatics/bts595

**Published:** 2012-10-09

**Authors:** Paul Kirk, Jim E. Griffin, Richard S. Savage, Zoubin Ghahramani, David L. Wild

**Affiliations:** ^1^Systems Biology Centre, University of Warwick, Coventry, CV4 7AL, ^2^School of Mathematics, Statistics and Actuarial Science, University of Kent, CT2 7NF and ^3^Department of Engineering, University of Cambridge, Cambridge, CB2 1PZ, UK

## Abstract

**Motivation:** The integration of multiple datasets remains a key challenge in systems biology and genomic medicine. Modern high-throughput technologies generate a broad array of different data types, providing distinct—but often complementary—information. We present a Bayesian method for the unsupervised integrative modelling of multiple datasets, which we refer to as MDI (Multiple Dataset Integration). MDI can integrate information from a wide range of different datasets and data types simultaneously (including the ability to model time series data explicitly using Gaussian processes). Each dataset is modelled using a Dirichlet-multinomial allocation (DMA) mixture model, with dependencies between these models captured through parameters that describe the agreement among the datasets.

**Results:** Using a set of six artificially constructed time series datasets, we show that MDI is able to integrate a significant number of datasets simultaneously, and that it successfully captures the underlying structural similarity between the datasets. We also analyse a variety of real *Saccharomyces cerevisiae* datasets. In the two-dataset case, we show that MDI’s performance is comparable with the present state-of-the-art. We then move beyond the capabilities of current approaches and integrate gene expression, chromatin immunoprecipitation–chip and protein–protein interaction data, to identify a set of protein complexes for which genes are co-regulated during the cell cycle. Comparisons to other unsupervised data integration techniques—as well as to non-integrative approaches—demonstrate that MDI is competitive, while also providing information that would be difficult or impossible to extract using other methods.

**Availability:** A Matlab implementation of MDI is available from http://www2.warwick.ac.uk/fac/sci/systemsbiology/research/software/.

**Contact:** D.L.Wild@warwick.ac.uk

**Supplementary information:** Supplementary data are available at *Bioinformatics* online.

## 1 INTRODUCTION

The wide range of modern high-throughput genomics technologies has led to a rapid increase in both the quantity and variety of functional genomics data that can be collected. For example, large-scale microarray (Lockhart *et al.*, 1996; Schena *et al.*, 1995), chromatin immunoprecipitation (ChIP) chip (Solomon *et al.*, 1988) and tandem affinity purification (Puig *et al.*, 2001; Rigaut *et al.*, 1999) datasets are available for a broad selection of organisms, providing measurements of mRNA expression, protein–DNA binding and protein–protein interactions (PPIs). In the forthcoming era of personal genomic medicine, we may reasonably expect genome sequences and other forms of high-throughput data (such as gene expression, alternative splicing, DNA methylation, histone acetylation and protein abundances) to be routinely measured for large numbers of people. The development of novel statistical and computational methodology for integrating diverse data sources is therefore essential, and it is with this that the present work is concerned.

As is common in statistics and machine learning, data integration techniques can be broadly categorized as either *supervised* (where a training/gold-standard set with known labels is used to learn statistical relationships) or *unsupervised* (where there is no training dataset, but we nevertheless seek to identify hidden structure in the observed data; e.g. by clustering). Our proposed method is unsupervised, but there are also a number of supervised learning algorithms that are designed to integrate multiple data sources; we now briefly mention these for the sake of completeness. These have proven highly successful in several contexts, often when predicting whether a link or interaction exists between two genes or proteins. Depending on the application, the link might represent (to provide just a few examples) protein–protein binding (Jansen *et al.*, 2003; Rhodes *et al.*, 2005), or a synthetic sick or lethal interaction (Wong *et al.*, 2004) or might indicate that the two genes have been implicated in the same biological process (Myers and Troyanskaya, 2007). Approaches for predicting these links often proceed by collecting a gold-standard set of positive and negative interactions (see, for contrasting examples, Jansen *et al.*, 2003; Lee *et al.*, 2004; Myers *et al.*, 2005), and then training statistical models (e.g. decision trees, naive Bayes classifiers) that predict the presence/absence of these interactions. These models may then be applied to predict the presence/absence of previously unknown interactions. Because training and prediction are performed on the basis of information collected from multiple different data sources, these approaches provide a form of data integration. Such supervised data integration techniques have proven highly effective for predicting interactions, some of which may then be verified experimentally (e.g. Rhodes *et al.*, 2005; Huttenhower *et al.*, 2009). Moreover, the work of Huttenhower *et al.* (2009) demonstrates that such approaches may be used to integrate whole-genome scale datasets. The Bayesian network approach of Troyanskaya *et al.* (2003) was a precursor to many of these supervised approaches, but differs from the others in that it uses knowledge from human experts to integrate predictions derived from diverse datasets.

Here we propose a novel *unsupervised* approach for the integrative modelling of multiple datasets, which may be of different types. For brevity, we refer to our approach as MDI, simply as a shorthand for ‘Multiple Dataset Integration’. We model each dataset using a Dirichlet-multinomial allocation (DMA) mixture model (Section 2.1), and exploit statistical dependencies between the datasets to share information (Section 2.2). MDI permits the identification of groups of genes that tend to cluster together in one, some or all of the datasets. In this way, our method is able to use the information contained within diverse datasets to identify groups of genes with increasingly specific characteristics (e.g. not only identifying groups of genes that are co-regulated, but additionally identifying groups of genes that are both co-regulated *and* whose protein products appear in the same complex).

Informally, our approach may be considered as a ‘correlated clustering’ model, in which the allocation of genes to clusters in one dataset has an influence on the allocation of genes to clusters in another. This contrasts with ‘simple’ clustering approaches (such as *k*-means, hierarchical clustering, etc) in which the datasets are clustered independently (or else concatenated and treated as a single dataset). It also clearly distinguishes our methodology from *biclustering* (e.g. Cheng and Church, 2000; Reiss *et al.*, 2006). Biclustering is the clustering of both dimensions in a single dataset (e.g. both genes and experiments in a gene expression dataset). MDI, in contrast, clusters a single dimension (e.g. genes) across multiple datasets. Biclustering is not applicable here as the datasets can be arbitrarily different, making any clustering across all features difficult. MDI avoids the problem of comparing different data types by instead learning the degree of similarity between the clustering structures (i.e. the gene-to-cluster allocations) in different datasets (Section 2.2).

MDI makes use of mixture models, which have become widespread in the context of unsupervised integrative data modelling (e.g. Barash and Friedman, 2002; Liu *et al.*, 2006, 2007), gaining increased popularity in recent years (Rogers *et al.*, 2010; Savage *et al.*, 2010). The principal advantages of using mixture models are as follows: (i) they provide flexible probabilistic models of the data; (ii) they naturally capture the clustering structure that is commonly present in functional genomics datasets; and (iii) by adopting different parametric forms for the mixture components, they permit different data types to be modelled (see also Section 2.1). An early application to data integration is provided by Barash and Friedman (2002), who performed integrative modelling of gene expression and binding site data.

As part of our approach, we infer parameters that describe the levels of agreement between the datasets. Our method may thus be viewed as extending the work of Balasubramanian *et al.* (2004). In this regard, MDI is also related to the approach of Wei and Pan (2012), which models the correlation between data sources as part of a method that classifies genes as targets or non-targets of a given transcription factor (TF) using ChIP–chip, gene expression and DNA binding data, as well as information regarding the position of genes on a gene network. Perhaps most closely related to MDI (in terms of application) are the methods of Savage *et al.* (2010) and *iCluster* (Shen *et al.*, 2009). Savage *et al.* (2010) adopt a mixture modelling approach, using a hierarchical Dirichlet process (DP) to perform integrative modelling of two datasets. As well as significant methodological differences, the principal practical distinction between this approach and MDI is that we are able to integrate more than two datasets, any or all of which may be of different types (Section 2). Like MDI, the *iCluster* method of Shen *et al.* (2009) permits integrative clustering of multiple (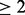) genomic datasets, but uses a joint latent variable model (for details, see Shen *et al.*, 2009). In contrast to MDI, *iCluster* seeks to find a single common clustering structure for all datasets. Moreover, *iCluster* must resort to heuristic approaches to estimate the number of clusters, whereas MDI infers this automatically (Section 2.1). We demonstrate that MDI provides results that are competitive with the two-dataset approach of Savage *et al.* (2010) in Section 3.2, and provide a comparison of results obtained using MDI, *iCluster* and simple clustering approaches in the Supplementary Material.

The potential biological applications of our approach are diverse, as there are many experimental platforms that produce measurements of different types, which might be expected to possess similar (but not necessarily identical) clustering structures. For example, in the two-dataset case, related methodologies have been used to discover transcriptional modules (Liu *et al.*, 2007; Savage *et al.*, 2010) and prognostic cancer subtypes (Yuan *et al.*, 2011) through the integration of gene expression data with TF binding (ChIP–chip) data and copy number variation data, respectively. A related approach was also used by Rogers *et al.* (2008) to investigate the correspondence between transcriptomic and proteomic expression profiles. In the example presented in this article, we focus on the biological question of identifying protein complexes whose genes undergo transcriptional co-regulation during the cell cycle.

The outline of this article is as follows. In Section 2, we briefly provide some modelling background and present our approach. Inference in our model is performed via a Gibbs sampler, which is provided in the Supplementary Material. In Section 3, we describe three case study examples, in all of which we use publicly available *Saccharomyces cerevisiae* (baker’s yeast) datasets. We present results in Section 4 and a discussion in Section 5.

## 2 METHODS

In this section, we provide some background regarding DMA mixture models (Section 2.1), and consider how these may be extended to allow us to perform integrative modelling of multiple datasets (Section 2.2). Inference in the resulting model (which we henceforth refer to as MDI) is performed using a Gibbs sampler (Supplementary Material). We briefly describe in Section 2.4 how the resulting posterior samples may be effectively summarized.

### 2.1 Dirichlet-multinomial allocation mixture models

We model each dataset using a finite approximation to a DP mixture model (Ishwaran and Zarepour, 2002), known as a DMA mixture model (Green and Richardson, 2001). Such models have the following general form:
(1)
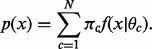



In the above, *p*(*x*) denotes the probability density model for the data, which is here an *N* component mixture model. The 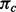
’s are mixture proportions, *f* is a parametric density (such as a Gaussian) and 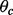 denotes the vector of parameters associated with the *c*-th component. Importantly, different choices for the density *f* allow us to model different types of data (for example, a normal distribution might be appropriate for continuous data, whereas a multinomial might be appropriate for categorical data).

Given observed data 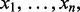 we wish to perform Bayesian inference for the unknown parameters in this model. As is common in mixture modelling (e.g. Dempster *et al.*, 1977; see also Friedman *et al.*, 2004 for a graphical model perspective), we introduce latent *component allocation* variables 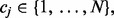 such that 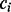 is the component responsible for observation 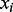. We then specify the model as follows:
(2)
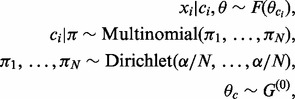

where *F* is the distribution corresponding to density *f*, 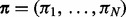 is the collection of *N* mixture proportions, 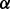 is a mass/concentration parameter (which may also be inferred) and 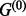 is the prior for the component parameters. Bayesian inference for such models may be performed via Gibbs sampling (Neal, 2000). Note that a realization of the collection of component allocation variables, 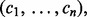 defines a *clustering* of the data (i.e. if 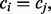 then 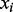 and 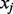 are clustered together). Because each 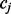 is a member of the set 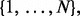 it follows that the value of *N* places an upper bound on the number of clusters in the data.

The DP mixture model may be derived by considering the limit 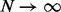 in Equation (1) (Neal, 1992; Rasmussen, 2000). In the present article, it is convenient to persist with finite *N* (Section 2.2). The important point is that *N* just places an upper bound on the number of clusters present in the data (because, as in the infinite DP case, not all of the components need to be ‘occupied’; i.e. not all components need to have observations associated with them), and hence *N* does not specify the precise number of clusters *a priori*. Provided *N* is taken sufficiently large, the number of clusters present in the data will be (much) less than *N*, and we will retain the ability to identify automatically the number of clusters supported by the data. Theoretical justifications for ‘large’ mixture models such as this (in which the number of components in the mixture is larger than the true number of clusters in the data) are provided by Rousseau and Mengersen (2011). A choice of *N* = *n* would set the upper bound on the number of clusters to be equal to the number of genes. As a tradeoff with computational cost, we take 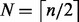 throughout this article.

### 2.2 Dependent component allocations

**Fig. 1.**
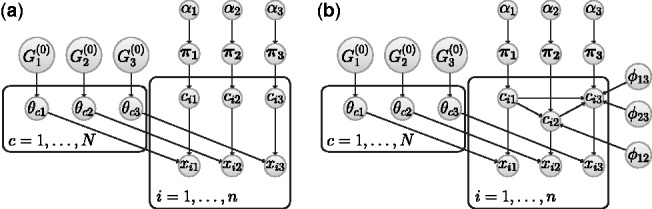
Graphical representation of three DMA mixture models. (**a**) Independent case. (**b**) The MDI model. In both (a) and (b), 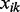 denotes the 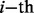 observation in dataset *k* and is generated by mixture component 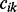. The prior probabilities associated with the distinct component allocation variables, 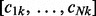, are given in the vector 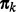, which is itself assigned a symmetric Dirichlet prior with parameter 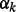. The parameter vector, 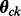, for component *c* in dataset *k* is assigned a 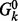 prior. In (b), we additionally have 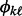 parameters, each of which models the dependence between the component allocations of observations in dataset *k* and 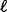

We are interested in the situation where we have a collection of *n* genes, for each of which we have measurements from *K* different data sources. One possible modelling approach would be to fit *K* independent DMA mixture models, represented graphically in Figure 1a for the case *K* = 3. However, this neglects to consider (and fails to exploit) structure within the data that may be common across some or all of the different sources. For example, a set of co-regulated genes might be expected to have similar expression profiles, as well as have a common collection of proteins that bind their promoters. We therefore propose a model in which we allow dependencies *between* datasets at the level of the component allocation variables, 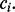

We consider *K* mixture models (one for each dataset), each defined as in Equations (1) and (2). We add right subscripts to our previous notation to distinguish between the parameters of the *K* different models (so that 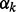 is the mass parameter associated with model *k*, etc.) and take 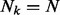 in all mixture models. Note that each model is permitted to have a different mass parameter, 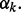 MDI links these models together at the level of the component allocation variables via the following conditional prior:
(3)
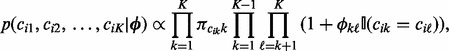

where 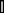 is the indicator function, 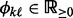 is a parameter that controls the strength of association between datasets *k* and 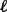, and 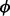 is the collection of all *K*(*K* − 1)/2 of the 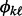
’s. For clarity, note that 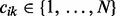 is the component allocation variable associated with gene *i* in model *k*, and that 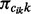 is the mixture proportion associated with component 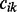 in model *k*. Informally, the larger 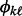, the more likely it is that 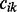 and 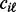 will be the same, and hence the greater the degree of similarity between the clustering structure of dataset *k* and dataset 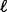. In Figure 1b, we provide a graphical representation of our model in the case *K* = 3. If all 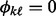, then we recover the case of *K*-independent DMA mixture models (Fig. 1a). Note that 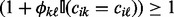, hence if 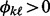 then we are up-weighting the prior probability that 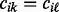 (relative to the independent case).

Linking the mixture models at the level of the component allocation variables provides us with a means to capture dependencies between the datasets in a manner that avoids difficulties associated with the datasets being of different types and/or having different noise properties.

An important feature of our model is that there is a correspondence between the component labels across the datasets. That is, our model implicitly ‘matches up’ Component *c* in Dataset *k* with Component *c* in Dataset 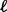. This allows us to identify groups of genes that tend to be allocated to the same component (i.e. which tend to cluster together) in multiple datasets (Section 2.4). It is this desire to ‘match up’ components across datasets that motivates our use of finite approximations to DP mixture models. Had we used an infinite mixture model, matching components across datasets would be more problematic. We reiterate that the finite *N* that appears in our mixture models merely places an upper bound on the number of clusters in each dataset (as not all components need to be occupied), and hence is not restrictive in practice. Note that while this upper bound is the same for each data set, the actual number of occupied components (i.e. clusters) is inferred separately for each dataset and in general will be different for each one.

### 2.3 Modelling different data types

To specify our model fully, we must provide parametric densities, *f*, appropriate for each data source. It is important to note that we may tailor our choice of *f* to reflect the data sources that we seek to model. In the present work, we use Gaussian process models (Cooke *et al.*, 2011; Kirk and Stumpf, 2009; Rasmussen and Williams, 2006) for gene expression time course data, and use multinomial models for categorical data (e.g. discretized gene expression levels). For comparison with the results of Savage *et al.* (2010), we also consider in our second example (Sections 3.2 and 4.2) a bag-of-words model for ChIP–chip data. Full details of all of these models are given in the Supplementary Material, where we also provide a Gibbs sampler for performing inference. As in Nieto-Barajas *et al.* (2004), posterior simulation for our model is aided by the strategic introduction of an additional latent variable (Supplementary Material for details).

### 2.4 Extracting fused clusters from posterior samples

We wish to identify groups of genes that tend to be grouped together in multiple datasets. Suppose we have a collection of *K* datasets, which we label as Dataset 1,…, Dataset *K*. We are interested in identifying groups of genes that tend to cluster together amongst some subcollection of the datasets. Let 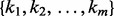 be a subset of 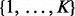. Our aim is to identify groups of genes that cluster together in all of Dataset 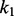,…, Dataset 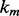. Adapting terminology from Savage *et al.* (2010), we define the probability of the *i*-th gene being *fused* across datasets 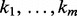 to be the posterior probability that 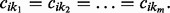 For brevity, we denote this posterior probability by 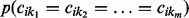. We calculate this quantity as the proportion of posterior samples for which 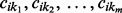 are all equal. We may clearly calculate these posterior fusion probabilities for any combination of the datasets (pairs, triplets, etc.), simply by considering the appropriate subset of 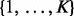. We say that the *i*-th gene is fused across datasets 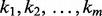 if 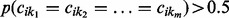, and we denote the set of all such fused genes by 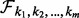.

If gene *i* is a member of 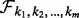, this simply tells us that the component allocation variables 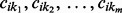 tend to be equal (i.e. gene *i* tends to be allocated to the same component across datasets 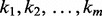). We also wish to identify the clustering structure that exists amongst these fused genes. From our Gibbs sampler, we have a collection of sampled component allocations for each member of 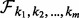. We identify a final clustering for the set of fused genes by searching amongst the sampled component allocations to find the one that maximizes the posterior expected adjusted Rand index (ARI; Fritsch and Ickstadt, 2009). The resulting *fused clusters* contain groups of genes that tend to cluster together across datasets 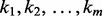.

## 3 EXAMPLES

To demonstrate the usage and utility of MDI, we consider three examples using publicly available *S. cerevisiae* datasets. We specify the priors adopted for unknown parameters and provide Markov chain Monte Carlo running specifications in the Supplementary Material. Each of our examples serves a different purpose. In the first (Section 3.1), we consider an easily interpretable synthetic dataset, which allows us to illustrate the types of results that can be obtained using MDI. In the second (Section 3.2), we seek to compare our method with the present state-of-the-art in data integration (namely, the approach of Savage *et al.*, 2010). Although this approach is limited to integrating two datasets only, it provides a useful benchmark for MDI. Finally, in Section 3.3, we provide an example that allows us to explore the benefits offered by MDI that go beyond the existing state-of-the-art. We consider the integration of three datasets, two of which comprise static measurements (ChIP–chip and PPI), and the other of which comprises gene expression time course data.

### 3.1 6-dataset synthetic example

**Fig. 2.**
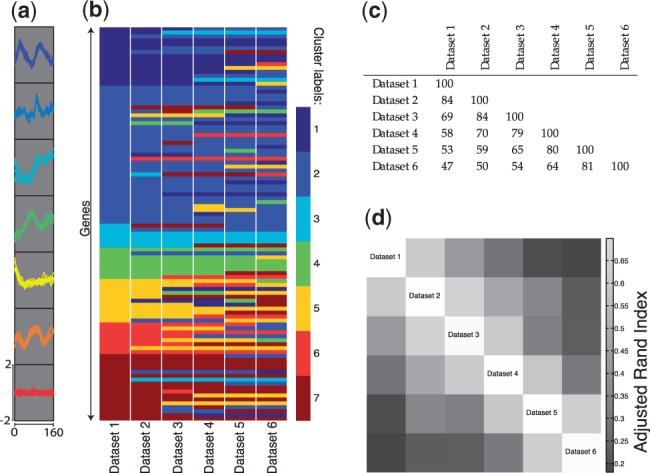
(**a**) The data for the six-dataset synthetic example, separated into seven clusters. (**b**) A representation of how the cluster labels associated with each gene vary from dataset to dataset. Genes are ordered so that the clustering of Dataset 1 is the one that appears coherent. (**c**) A table showing the number of genes having the same cluster labels in datasets *i* and *j*. (**d**) A heatmap depiction of the similarity matrix formed by calculating the ARI between pairs of datasets

To illustrate the properties of our model, we start with a six-dataset synthetic example. Dataset 1 is constructed by taking a 100-gene subset of the gene expression time course data of Cho *et al.* (1998), and may be partitioned into seven easily distinguishable clusters (Fig. 2a). We therefore associate with each time course a cluster label, 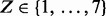. For 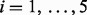, we form Dataset *i* + 1 by randomly selecting 25 time courses from Dataset *i* and randomly permuting their associated gene names (but not their cluster labels). Thus, for a maximum of 25 genes, the cluster label associated with gene *g* in Dataset *i* may be different from the cluster label associated with the same gene in Dataset *i* + 1. Figure 2b and c further illustrate this dataset. A formal approach for comparing the allocation of genes to clusters is to calculate the ARI between each pair of clustering partitions (Hubert and Arabie, 1985; Rand, 1971). Figure 2d provides a heatmap depiction of the similarity matrix formed by calculating pairwise ARIs.

### 3.2 Integrating expression and ChIP data

To compare our method with an existing approach for unsupervised data integration, we apply MDI to an example previously considered by Savage *et al.* (2010) in the context of transcriptional module discovery. We take expression data from a 205-gene subset of the galactose-use data of Ideker *et al.* (2001), which we integrate with ChIP–chip data from Harbison *et al.* (2004). The expression data were discretized, as in Savage *et al.* (2010). The 205 genes appearing in this dataset were selected in Yeung *et al.* (2003) to reflect four functional Gene Ontology (GO) categories. Although this functional classification must be used with some degree of caution (Yeung *et al.*, 2003), it provides a reasonable means by which to validate the groupings defined by our method. We use the same version of the Harbison *et al.* dataset as considered by Savage *et al.* (2010) (significance threshold *P* = 0.001), which provides binding information for 117 transcriptional regulators. For brevity, we henceforth refer to the data of Harbison *et al.* as ‘ChIP data’, although we emphasise that this dataset comprises measurements corresponding to a compendium of 117 TFs, rather than to a single particular TF. Discretizing the data (both expression and ChIP–chip) might seem like an unnecessary simplification (as our model can accommodate continuous static measurements through an appropriate choice of component density function, *f*), but it helps to ensure that our comparison to the results of Savage *et al.* (2010) is fair. Moreover, discretization of the ChIP data simplifies modelling and interpretation of the data (the *ij*-entry of our ChIP data matrix is 1 if we have high confidence that 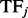 is able to bind the promoter region of gene *i*, and 0 otherwise), although we acknowledge that this is likely to incur some small information loss.

### 3.3 Integrating expression, ChIP and PPI data

For an example with three diverse data types, we integrate the ChIP data of Harbison *et al.* with binary PPI data obtained from BioGRID (Stark *et al.*, 2006) and a gene expression time course dataset of Granovskaia *et al.* (2010), with the initial intention of identifying protein complexes whose genes undergo transcriptional co-regulation during the cell cycle. We consider the Granovkskaia *et al.* cell cycle dataset that comprises measurements taken at 41 time points, and which was obtained from cells synchronized using alpha factor arrest. We considered only genes identified in Granovskaia *et al.* (2010) as having periodic expression profiles. After removing those for which there was no ChIP or PPI data, we were left with 551 genes. Our binary PPI data matrix then has rows indexed by these 551 genes, and columns indexed by all of the proteins for which physical interactions identified via yeast 2-hybrid or affinity capture assays have been reported in BioGRID. The *ij*-entry of the PPI data matrix is 1 if there is a reported interaction between protein *j* and the protein product of gene *i* (and 0 otherwise). In an effort to reduce the number of uninformative features, we removed columns containing fewer than five 1s, leaving 603 columns.

## 4 RESULTS

### 4.1 6-dataset synthetic example

**Fig. 3.**
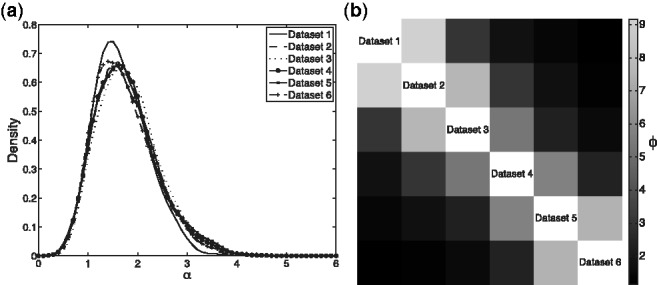
(**a**) Densities fitted to the sampled values of 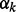. (**b**) Heatmap representation of the matrix with 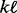-entry 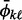, the posterior mean value for 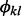

Figure 3a shows estimated posterior densities for the mass parameters, 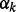 (obtained from the samples generated by our Gibbs sampler using kernel density estimation). Because each of our datasets is identical (up to permutation of gene names), these distributions should be close to identical, as is the case. For each pair of datasets, we used the posterior 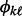 samples to estimate posterior means, 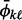. We used these to form a similarity matrix whose 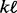-entry is 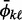 (with 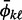 defined to be 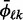 whenever 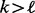, and with 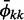 left undefined). This is shown as a heatmap in Figure 3b. Although they do so in different ways, both the ARI and the dataset association parameters quantify the degree of similarity between the allocation of genes to clusters in pairs of datasets. The similarity of Figures 2d and 3b is therefore reassuring.

To test our ability to identify fused genes, we calculated pairwise fusion probabilities, 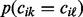, for each gene *i* and each pair of datasets 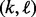. If the true cluster label of gene *i* is the same in datasets *k* and 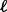, then 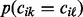 should be high (>0.5) so that the gene may be correctly identified as fused. Across all pairs of datasets, the minimum pairwise fusion probability for such genes was 0.90 and the mean was 0.97. Conversely, for genes having *different* cluster labels in datasets *k* and 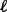, the maximum pairwise fusion probability was 0.05 and the mean was 0.01. Because our fusion threshold is 0.5, we are in this case able to identify the fusion status correctly for all genes.

### 4.2 Expression + ChIP example

We ran MDI using a multinomial likelihood model for both the discretized expression data and the binary ChIP–chip data. We estimated pairwise fusion probabilities and extracted fused clusters, as described in Section 2.4. We identified 52 fused genes, grouped into three clusters. We compared these clusters to the functional classes defined in Yeung *et al.* (2003). Within each cluster, all genes had the same functional classification, whereas genes in different clusters possessed different classifications.

In Savage *et al.* (2010), a bag-of-words model was used to model TF binding data. To permit a fair comparison of the two approaches, we therefore re-ran MDI using a bag-of-words likelihood model for the ChIP data. Following Savage *et al.* (2010), we then calculated the Biological Homogeneity Index (BHI; Datta and Datta, 2006) for the resulting fused clusters. To calculate the BHI scores, we used the R package clValid (Brock *et al.*, 2008) together with the GO annotations in the org.Sc.sgd.db Bioconductor package (Carlson *et al.*, 2010). The clValid package provides four different BHI scores, depending on which GO functional categories are used to define the set of annotations. All categories may be considered or just one of biological process (bp), cellular component (cc) and molecular function (mf). We report all four BHI scores in Table 1, for the fused clusters defined by (i) the method of Savage *et al.* (2010); (ii) MDI using a bag-of-words likelihood and (iii) MDI using a multinomial likelihood. The BHI scores for MDI (bag-of-words) and the method of Savage *et al.* (2010) are almost identical, although MDI (bag-of-words) identifies a greater number of fused genes.

**Table 1.** BHI scores for the fused clusters obtained using the method of Savage *et al.* (2010), together with those obtained using MDI

Method	BHI (all)	BHI (bp)	BHI (mf)	BHI (cc)	Number of genes
Savage *et al.* (2010)	0.98	0.85	0.71	0.98	72
MDI (bag-of-words)	0.98	0.85	0.72	0.97	172
MDI (multinomial)	1.00	0.89	0.77	1.00	52

### 4.3 Expression + ChIP + PPI example

We applied MDI to the example of Section 3.3 (using GP models for the gene expression time courses, and multinomial models for the ChIP and PPI datasets), to identify groups of genes that are co-regulated during the yeast cell cycle, and whose protein products appear in the same complex. We identified genes fused across all three datasets, as well as genes fused across pairs of datasets. We then determined the fused clusters for each of these combinations (Section 2.4). Additionally, we identified clusters for the ‘single dataset fusion’ case (which amounts to identifying a single clustering partition for each of our three datasets considered separately). We assess the quality of our clusterings using GO Term Overlap (GOTO) scores (Mistry and Pavlidis, 2008). These assign a score to a pair of genes according to how many GO terms they have in common. This contrasts with BHI, which just assigns a score of 0 or 1 to gene pairs depending on whether or not they share a common GO term. The GOTO scores therefore provide a more finely grained assessment, which implicitly takes into account the hierarchical structure of the GO. This is invaluable here because (as a result of selecting only genes found to have periodic expression profiles during the cell cycle) any two randomly selected genes are likely to share some high-level GO terms (see the Supplementary Material for more details). The GOTO scores are reported in Table 2

The GOTO scores generally increase as we require agreement across more datasets, while the number of fused genes decreases. Note that this decrease is simply a consequence of requiring agreement among a larger collection of datasets. For example, as the set 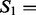 {genes that are co-regulated *and* have protein products that appear in the same complex} is a subset of 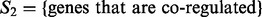, it is inevitable that the number of genes of the former type will be less than or equal to the number of genes of the latter type. In other words, requiring agreement across multiple datasets enables us to identify clusters of genes that have increasingly specific shared characteristics. This is reflected in the increasing GOTO scores, which indicate that genes in the same cluster tend to share a greater number of lower-level (more specific) GO terms.

**Fig. 4.**
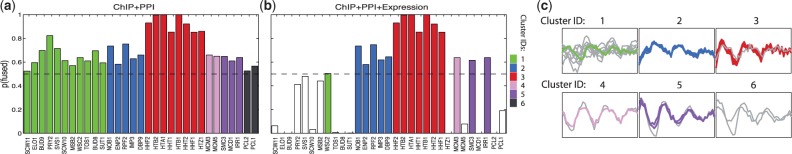
(**a**) Pairwise fusion probabilities for the 31 genes identified as fused across the ChIP and PPI datasets in the ‘Expression + ChIP + PPI’ example. Colours correspond to fused clusters and the dashed line indicates the fusion threshold. (**b**) Three-way fusion probabilities for the same 31 genes. Genes that do not exceed the fusion threshold have white bars. (**c**) The expression profiles for genes identified as fused according to the ChIP and PPI datasets. The coloured lines indicate genes that are also fused across the expression dataset as well

In Figure 4, we compare the clusters formed by the genes fused across all three datasets with those formed by the genes fused across just the PPI and ChIP datasets. Figure 4a and b illustrate fusion probabilities for the 31 genes identified as fused across the PPI and ChIP datasets. Each bar in Figure 4a corresponds to a particular gene (as labelled), and represents the posterior probability of that gene being fused across the ChIP and PPI datasets. The corresponding bar in Figure 4b represents the probability of the gene being fused across all three datasets. Figure 4c shows the expression profiles for genes identified as fused across the PPI and ChIP datasets, with genes fused across all three datasets shown in colour. Supplementary Figure 2 further illustrates the fused clusters, whereas Table 3 shows the fused cluster labels and provides descriptions for the genes fused across all three datasets.

**Table 2.** GOTO scores for fused clusters obtained for all combinations of the expression, ChIP and PPI datasets

Dataset(s)	GOTO (bp)	GOTO (mf)	GOTO (cc)	Number Of genes
ChIP	6.36	0.97	8.53	551
PPI	11.04	1.51	11.11	551
Expression	7.66	1.15	9.48	551
ChIP + PPI	27.04	3.47	18.99	31
ChIP + Expression	24.46	2.93	16.87	48
PPI + Expression	26.04	3.69	22.35	32
ChIP + PPI + Expression	34.81	2.46	26.70	16

**Table 3.** Clusters formed by the genes fused across all 3 datasets

ID	Gene	Brief description
2	*NOB1*	Involved in synthesis of 40S ribosomal subunits
2	*ENP2*	Required for biogenesis of the small ribosomal subunit
2	*RPF2*	Involved in assembly of 60S ribosomal subunit
2	*IMP3*	Component of the SSU processome
2	*DBP9*	Involved in biogenesis of 60S ribosomal subunit
3	*HHF2*	Histone H4, core histone protein
3	*HTB2*	Histone H2B, core histone protein
3	*HTA1*	Histone H2A, core histone protein
3	*HHT1*	Histone H3, core histone protein
3	*HTB1*	Histone H2B, core histone protein
3	*HHT2*	Histone H3, core histone protein
3	*HHF1*	Histone H4, core histone protein
5	*SMC3*	Subunit of the cohesin complex
5	*IRR1*	Subunit of the cohesin complex

Descriptions were derived from the *Saccharomyces* Genome Database (Cherry *et al.*, 1998). The IDs in this table correspond to the cluster IDs in Figure 4, with singletons omitted.

We can see from Figure 4a and b that the integration of the expression data in addition to the ChIP and PPI data results in Cluster 1 (green) and Cluster 6 (black) being effectively removed. Although many of the genes in Cluster 1 are annotated as cell wall proteins (Supplementary Material), and although the two genes in Cluster 6 are both cyclins, the genes within these clusters have different expression patterns to one another (Fig. 4c, panels 1 and 6). Genes are also lost from Clusters 4 and 5 (shown pink and purple). However, further analysis suggests that this is owing to data normalization effects (Supplementary Material). Cluster 2 (blue) is robust to the additional inclusion of expression data, indicating that there is no significant disagreement amongst the three datasets regarding the existence of this cluster. Cluster 3 (red) is also relatively robust, with only one less gene when we consider the fusion of all three datasets, compared to the fusion of just the ChIP and PPI datasets (Fig. 4a and b). We note that the genes in Clusters 2 and 3 all have key roles, either encoding core histone proteins or being involved in ribosome biogenesis (Table 3).

Interestingly, the gene lost from Cluster 3 (the histone cluster) is HTZ1, which encodes the variant histone H2A.Z (Jackson *et al.*, 1996; Santisteban *et al.*, 2000). The function of H2A.Z is different to that of the major H2As (e.g. Jackson and Gorovsky, 2000). We can see from Figure 4c (panel 3) that the expression of this gene (shown grey) is subtly different to the expression of others in the cluster.

### 4.4 Comparison to other methods

In Section G of the Supplementary Material, we provide a comparison of MDI with other clustering methods, both in terms of performance and the types of results that can be obtained. The key properties of MDI that distinguish it from other clustering methods are (i) the clustering of genes in dataset *k* influences (and is influenced by) the clustering in dataset 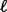, to an extent determined by the inferred 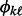 parameter; (ii) each dataset is permitted to have a different clustering structure (so each dataset may, for example, have a different number of clusters); (iii) the number of clusters is determined automatically as part of the inference procedure and (iv) there is a correspondence between the cluster labels in different datasets, which enables us to identify clusters of genes that exist across some or all of the datasets. Simple clustering methods (such as *k*-means and hierarchical clustering) can be used to cluster each of the datasets independently, but do not model the dependence/similarity between clustering structures in different datasets and do not enable clusters that exist across multiple datasets to be identified automatically. More sophisticated methods such as *iCluster* (Shen *et al.*, 2009) often share some of MDI’s properties, but do not allow for the identification of subsets of genes that cluster together across multiple datasets. The results of Section G of the Supplementary Material demonstrate that the ability to share information across datasets typically provides improvements in clustering quality, while MDI's additional ability to pick out clusters that exist across multiple datasets permits the identification of groups of genes with specific shared characteristics. Increasing the number of datasets across which we seek agreement in cluster assignment has the effect of increasing the specificity of these shared characteristics (which typically reduces the size of the gene subset—see Section 4.3 for further explanation).

### 4.5 Scaling and run-times

For typical examples (where the number of datasets, *K*, is relatively small), the scaling of MDI will be *O*(*KNn*) (see Supplementary Section D.5 for further details and specific run-times). MDI is particularly appropriate for applications in which a gene pre-selection step is performed (e.g. on the basis of differential expression). We anticipate applications to collections of 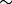
5 datasets, each comprising 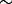
1000 genes. Parallelizing MDI using an approach such as the one described by Suchard *et al.* (2010) should be possible, and we are currently investigating this.

## 5 DISCUSSION

We have presented MDI, a novel Bayesian method for the unsupervised integrative modelling of multiple datasets. We have established that MDI provides competitive results with an existing method for integrating two datasets (Section 4.2), and is also able to integrate collections of more than two datasets (Sections 4.1 and 4.3). Our application to a three-dataset example (Section 4.3) demonstrated that requiring agreement across multiple datasets of different types can enable us to identify clusters of genes with increasingly specific shared characteristics. Moreover, we have found that sharing information across multiple datasets can improve cluster quality.

MDI adopts a modelling approach distinctly different from those adopted by existing integrative modelling methods. For example, the model of Savage *et al.* (2010) performs integrative modelling of two datasets only, achieved by introducing a ‘fused context’ (in which the two datasets are modelled together via a product of likelihoods) in addition to two ‘unfused contexts’ in which the two datasets are modelled separately. This is analogous to introducing—and modelling—an additional dataset. In contrast, MDI introduces just a single parameter, 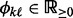, for each pair of datasets (Section 2.2), and it is this that provides MDI with the flexibility to perform integrative modelling of *multiple* datasets. The scalability of MDI may be further improved through parallelization of the type described by Suchard *et al.* (2010). This is an important direction for future work.

## Supplementary Material

Supplementary Data
