# Supplementary material for: Bayesian correlated clustering to integrate multiple datasets
Source: Bioinformatics. 2012 Oct 9;28(24):3290–7. doi: 10.1093/bioinformatics/bts595 (PMC3519452; doi:10.1093/bioinformatics/bts595)
Supplement: Supplementary Data [file supp_28_24_3290__index.html]

Bayesian correlated clustering to integrate multiple datasets — Supplementary Data 

# Bayesian correlated clustering to integrate multiple datasets

## Supplementary Data

files

**Files in this Data Supplement:**

- Supplementary Data - pdf file
