# Supplementary material for: Bayesian correlated clustering to integrate multiple datasets
Source: Bioinformatics. 2012 Oct 9;28(24):3290–7. doi: 10.1093/bioinformatics/bts595 (PMC3519452; doi:10.1093/bioinformatics/bts595)
Supplement: Supplementary Data [file supp_bts595_bts595_Bioinformatics_MDI_SupplementaryMaterial.pdf]

Bayesian integration of multiple datasets SUPPLEMENTARY MATERIAL

Paul Kirk[1], Jim E. Griffin[2], Richard S. Savage[1], Zoubin Ghahramani[3], and David L. Wild[1]

[1] Systems Biology Centre, University of Warwick, Coventry, CV4 7AL, UK
[2] School of Mathematics, Statistics and Actuarial Science, University of Kent, Canterbury, CT2 7NF, UK
[3] Department of Engineering, University of Cambridge, Cambridge CB2 1PZ, UK
D.L.Wild@warwick.ac.uk

Abstract. In this supplementary material, we include a number of results that were omitted from the main paper for the sake of brevity. In Section A, we provide full details of how inference may be performed for the MDI (Multiple Dataset Integration) model via a Gibbs sampler, and specify the priors used when considering the case study examples of the main paper. In Section B, we provide some supplementary figures for which there was insufficient room in the main text. In Section C we describe the probabilistic models that were used for each different data type, and then provide MCMC running specifications in Section D, together with some information on run times and the scaling of the algorithm. In Section E we provide further details regarding GO Term Overlap and in Section F we compare MDI to some simple clustering approaches, as well as to the integrative clustering *iCluster* algorithm. In Sections H and I, we elaborate upon the results presented in Section 4.3 of the main paper. First, in Section H, we present results for the 3 pairwise comparisons (namely, ChIP+PPI, ChIP+Expression and PPI+Expression). Then, in Section I, we explore the effects of data normalisation upon some of our results. Finally, in Section J, we extend the results of Section 4.2 of the main paper (the Expression+ChIP example), by additionally integrating a protein-protein interaction dataset.

A Inference in MDI

In this section, we provide full details of how inference may be performed for our model (via a Gibbs sampler), and specify the priors that we used in order to obtain the results presented in the main paper. We use the notation that was introduced in the main paper.

A.1 Model

We define the general model,

$$p(c_{i1}, c_{i2}, \ldots, c_{iK}|\boldsymbol{\phi}) \propto \prod_{k=1}^{K} \gamma_{c_{ik}k} \prod_{k=1}^{K-1} \prod_{\ell=k+1}^{K} \left(1 + \phi_{k\ell}\mathbb{I}(c_{ik} = c_{i\ell})\right), \tag{1}$$

where $\gamma_{1k}, \gamma_{2k}, \ldots, \gamma_{Nk} \overset{i.i.d.}{\sim} \text{Ga}(\alpha_k/N, 1)$, where "Ga" denotes the Gamma distribution. Writing $\pi_{ik} = \frac{\gamma_{ik}}{\sum_{j=1}^{N}\gamma_{jk}}$, we then have $\pi_{1k}, \ldots, \pi_{Nk} \sim \text{Dirichlet}(\alpha_k/N, \ldots, \alpha_k/N)$.

Recall that k is the index on the datasets, and that we have K datasets in total. We permit α_k to be different for each dataset. It follows that the gamma priors for $\gamma_{1k}, \ldots, \gamma_{1k}$ will in general have different shape parameters for each dataset, and so the $\text{Dirichlet}(\alpha_k/N, \ldots, \alpha_k/N)$ priors will have different mass parameters.

A.2 Normalising constant

It is straightforward to write down the normalising constant, Z, for Equation (1) above, simply by summing over all possibilities for the c_{ik}'s:

$$Z = \sum_{j_1=1}^{N}\sum_{j_2=1}^{N}\cdots\sum_{j_K=1}^{N}\left(\prod_{k=1}^{K}\gamma_{j_k k}\prod_{k=1}^{K-1}\prod_{\ell=k+1}^{K}\left(1 + \phi_{k\ell}\mathbb{I}(j_k = j_\ell)\right)\right). \tag{2}$$

The joint density for n genes is hence,

$$p(\{c_{i,1}, c_{i,2}, \ldots, c_{i,K}\}_{i=1}^{n}) = \frac{1}{Z^n} \prod_{i=1}^{n} \left(\prod_{k=1}^{K} \gamma_{c_{ik}k} \prod_{k=1}^{K-1} \prod_{\ell=k+1}^{K} (1 + \phi_{k\ell} \mathbb{I}(c_{ik} = c_{i\ell})) \right). \tag{3}$$

As in Nieto-Barajas *et al.* (2004), we introduce a strategic latent variable, v, such that,

$$p(\{c_{i,1}, c_{i,2}, \ldots, c_{i,K}\}_{i=1}^{n}, v) = \frac{v^{n-1} \exp(-vZ)}{(n-1)!} \times \prod_{i=1}^{n} \left(\prod_{k=1}^{K} \gamma_{c_{ik}k} \prod_{k=1}^{K-1} \prod_{\ell=k+1}^{K} (1 + \phi_{k\ell} \mathbb{I}(c_{ik} = c_{i\ell})) \right), \tag{4}$$

where Z is as given in Equation (2).

A.3 Conditionals

We find conditionals by inspection of the joint density given in Equation (4). This enables us to perform inference in our model via Gibbs sampling.

Conditional for v The conditional distribution for v is,

$$\mathrm{Ga}(n, Z).$$

Conditional for $\gamma_{j_m m}$ The conditional is $\mathrm{Ga}(a_\gamma, b_\gamma)$, where

$$a_\gamma = 1 + \sum_{i=1}^{n} \mathbb{I}(c_{im} = j_m),$$

and,

$$b_\gamma = v \sum_{j_1=1}^{N} \cdots \sum_{j_{m-1}=1}^{N} \sum_{j_{m+1}=1}^{N} \cdots \sum_{j_K=1}^{N} \left(\prod_{k=1;k\neq m}^{K} \gamma_{j_k k} \prod_{k=1}^{K-1} \prod_{\ell=k+1}^{K} (1 + \phi_{k\ell} \mathbb{I}(j_k = j_\ell)) \right).$$

Conditional for ϕ_{mp} The conditional is $\mathrm{Ga}(a_\phi, b_\phi)$, where

$$a_\phi = 1 + \sum_{i=1}^{n} \mathbb{I}(c_{im} = c_{ip}),$$

and,

$$b_\phi = v \sum_{j_m=j_p=1}^{N} \sum_{j_1=1}^{N} \cdots \sum_{j_{m-1}=1}^{N} \sum_{j_{m+1}=1}^{N} \cdots \sum_{j_{p-1}=1}^{N} \sum_{j_{p+1}=1}^{N} \cdots \sum_{j_K=1}^{N} \left(\prod_{k=1}^{K} \gamma_{j_k k} \prod_{k=1}^{K-1} \prod_{\ell=k+1;\ell\neq p}^{K} (1 + \phi_{k\ell} \mathbb{I}(j_k = j_\ell)) \prod_{k=1;k\neq m}^{p-1} (1 + \phi_{kp} \mathbb{I}(j_k = j_p)) \right).$$

Conditional for c_{im} Let x_{im} be the observation for gene i in dataset m. Define $x^c_{-i,m}$ to be the set of all observations (not including x_{im}) currently associated with component c in dataset m. Also define $c_{-i,m}$ to be the collection of c_{jm} for which $i \neq j$. Similarly, define $c_{i,-m}$ to be the collection of c_{ik} for which $k \neq m$. The conditional for c_{im} is then:

$$p(c_{im} = c|\boldsymbol{\phi}, c_{-i,m}, c_{i,-m}, x_{im}, x^c_{-i,m}) = b\gamma_{cm}\times \prod_{k=1}^{m-1}(1+\phi_{km}(\mathbb{I}(c_{ik}=c_{im}))) \prod_{k=m+1}^{K}(1+\phi_{mk}(\mathbb{I}(c_{im}=c_{ik}))) \int f_m(x_{im}, x^c_{-i,m}|\theta_m) g_m^{(0)}(\theta_m) d\theta_m.$$

Here, f_m is the likelihood model associated with dataset m, $g_m^{(0)}$ is the prior density associated with dataset m for the component parameters θ_m, and b is a normalising constant that ensures that,

$$\sum_{c=1}^{N} p(c_{im} = c|\boldsymbol{\phi}, c_{-i,m}, c_{i,-m}, x_{im}, x^c_{-i,m}) = 1.$$

A.4 Priors

We assume a $\text{Ga}(1, 0.2)$ prior for all ϕ_{mp} parameters. The prior on the $\gamma_{j_m m}$ parameters is $\text{Ga}(\alpha_m/N, 1)$. We infer the α_m's (the dataset-specific mass parameters) as part of our inference procedure, employing a Metropolis-Hastings step at the end of each complete Gibbs iteration. For the first and second examples in the main paper (Sections 3.1 and 3.2), we adopt $\text{Ga}(2, 4)$ priors for all α_m's. For the third example (where there are more genes), we adopt $\text{Ga}(2, 2)$ priors.

B Additional supplementary figures

B.1 Graphical model

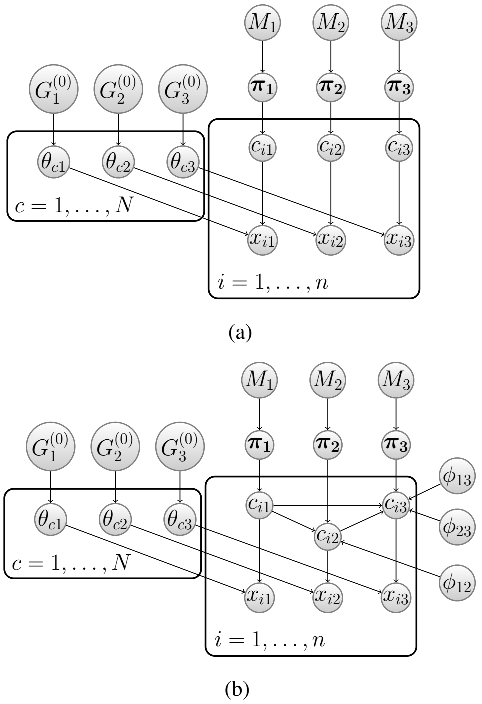

Fig. 1: Enlarged version of Figure 1 from the main paper. Graphical representation of $K = 3$ DMA mixture models. (a) Independent case. (b) Modelling dependence between the latent component allocation variables (the MDI model).

B.2 Illustration of the clusters formed by the genes fused over all 3 datasets in the Expression + ChIP + PPI example

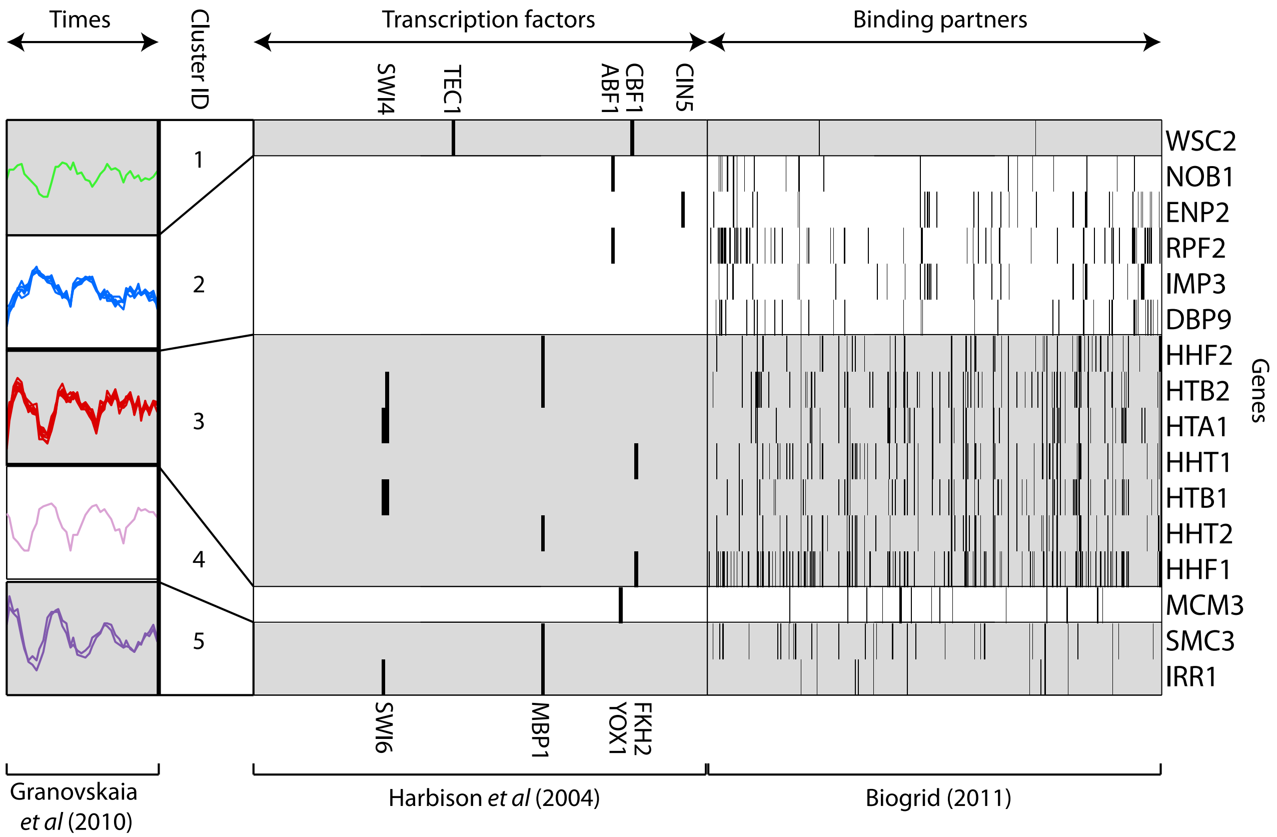

Fig. 2: Representation of the clusters formed by the genes fused across all 3 datasets. For the ChIP and PPI datasets, vertical black lines indicate binding.

C Probability models for different datasets

As stated in the main paper, in order to complete the specification of the model, we must provide details of the probability models used for the various data types. In this section, we provide details of the Gaussian process models used for the time course datasets, and the multinomial and bag-of-words models used for the categorical datasets.

C.1 Gaussian process model

We adopted Gaussian process (GP) models for the time course datasets (see, for example Kirk and Stumpf, 2009; Cooke *et al.*, 2011). We employed a squared exponential covariance function,

$$k_{SE}(t_i, t_j) = \sigma_f^2 \exp\left(-(t_i - t_j)^2/2l^2\right) + \sigma_\epsilon^2 \delta_{ij},$$

where δ_{ij} is the Kronecker delta function, t_i and t_j are time points, and σ_f, l and σ_ϵ are hyperparameters. Different components of the mixture model have different hyperparameters. To infer the hyperparameters, we employ a Metropolis-Hastings step every q-th Gibbs iteration (we take $q = 1$ for the synthetic example, and $q = 5$ for the "Expression + ChIP + PPI" example). In practice, we perform inference for the log of each hyperparameter, to sidestep positivity constraints. We adopt standard normal priors for each log-hyperparameter.

C.2 Multinomial model

We adopted multinomial models as the default choice for categorical datasets (e.g. discretised gene-expression [as in Section 3.2], ChIP-chip data [as in Sections 3.2 and 3.3], and PPI data [as in Section 3.3]). We describe the multinomial model below.

Suppose, for each gene, that we have measurements taken on Q features. Observations on each of these features take a value r from the set $\{1, \ldots, R\}$. For genes within a given cluster, we denote by x_{rq} the number of times we observe that the q-th feature takes value r, and we let $\mathbf{x}_q = [x_{1q}, \ldots, x_{Rq}]$. We denote the cluster-specific probability of getting value r for feature q by θ_{rq}, so that $\sum_{r=1}^{R} \theta_{rq} = 1$ and,

$$p(\mathbf{x}_q | \theta_{1q}, \ldots, \theta_{Rq}) = \prod_{r=1}^{R} \theta_{rq}^{x_{rq}}.$$

We adopt a Dirichlet$(\beta_{1q}, \ldots, \beta_{Rq})$ prior for $\theta_{1q}, \ldots, \theta_{Rq}$. Exploiting conjugacy of the Dirichlet and multinomial distributions, we may marginalise the unknown θ_{rq}'s to obtain:

$$p(\mathbf{x}_q | \beta_{1q}, \ldots, \beta_{Rq}) = \frac{\Gamma(B_q)}{\Gamma(S_q + B_q)} \prod_{r=1}^{R} \frac{\Gamma(x_{rq} + \beta_{rq})}{\Gamma(\beta_{rq})}$$

where $B_q = \sum_{r=1}^{R} \beta_{rq}$ and $S_q = \sum_{r=1}^{R} x_{rq}$. Assuming independence between features, we obtain the following (marginal) likelihood function:

$$f(\mathbf{x}_1, \ldots, \mathbf{x}_Q | \{\beta_{rq}\}_{r=1,\ldots,R; q=1,\ldots,Q}) = \prod_{q=1}^{Q} \frac{\Gamma(B_q)}{\Gamma(S_q + B_q)} \prod_{r=1}^{R} \frac{\Gamma(x_{rq} + \beta_{rq})}{\Gamma(\beta_{rq})}.$$

We set the Dirichlet prior hyperparameters, β_{rq}, to be 0.5.

C.3 Bag-of-words model

In our second example (Section 3.2), we considered a bag-of-words model for the binary ChIP-chip data (in order to facilitate comparison with the method of Savage *et al.*, 2010) in addition to our default multinomial model. We describe this below.

Suppose, for each gene, we have binary observations for each of Q features. Given a cluster of genes, we can count, for $q = 1, \ldots, Q$, the total number of genes in the cluster for which the observed value of feature q is a 1. Let x_q be the number of genes in the cluster for which the q-th feature is 1. We summarise the data in the cluster as a vector of counts, $\mathbf{x} = [x_1, \ldots, x_Q]$. Let $S = \sum_{q=1}^{Q} x_q$. We make the simplifying assumption that $\mathbf{x}$ was obtained via a multinomial experiment with S independent trials and Q possible outcomes. We denote the probability of outcome q by θ_q, so that:

$$p(\mathbf{x} | \theta_1, \ldots, \theta_Q) = \prod_{q=1}^{Q} \theta_q^{x_q}.$$

We adopt a Dirichlet$(\beta_1, \ldots, \beta_Q)$ prior for the unknown θ_q. Exploiting conjugacy of the Dirichlet and multinomial distributions, we may marginalise the unknown θ_q's to obtain the following (marginal) likelihood function:

$$f(\mathbf{x} | \beta_1, \ldots, \beta_Q) = \frac{\Gamma(B)}{\Gamma(S + B)} \prod_{q=1}^{Q} \frac{\Gamma(x_q + \beta_q)}{\Gamma(\beta_q)}$$

where $B = \sum_{q=1}^{Q} \beta_q$.

We set the Dirichlet prior hyperparameters, β_q, to be 0.5.

D MCMC running specifications

In this section, we provide the MCMC running specifications for each of the examples.

D.1 Synthetic and Expression+ChIP examples

We ran 20 chains in parallel, obtaining 10,000 samples from each. We removed the first 50% as burn-in, and thinned the remaining samples by only retaining every 10-th sample. We then combined the resulting 20 sets of 500 samples.

D.2 Expression+ChIP+PPI example

We ran 10 chains in parallel, obtaining 8,500 samples from each. We removed the first 6,000 as burn-in, and thinned the remaining samples by only retaining every 5-th sample. We then combined the resulting 10 sets of 500 samples.

D.3 Diagnostic plots I - The number of clusters at each iteration

We monitored the mixing of the MCMC chains by recording the number of clusters (i.e. the number of occupied components) at each iteration. This information is illustrated in Figures 3 – 5. Note that we plot only the thinned samples, and that the burn-in period is indicated in the first plot of each figure. In all cases, mixing occurs relatively rapidly, and our burn-in appears conservative (i.e. a shorter burn-in would usually have been acceptable).

Synthetic example

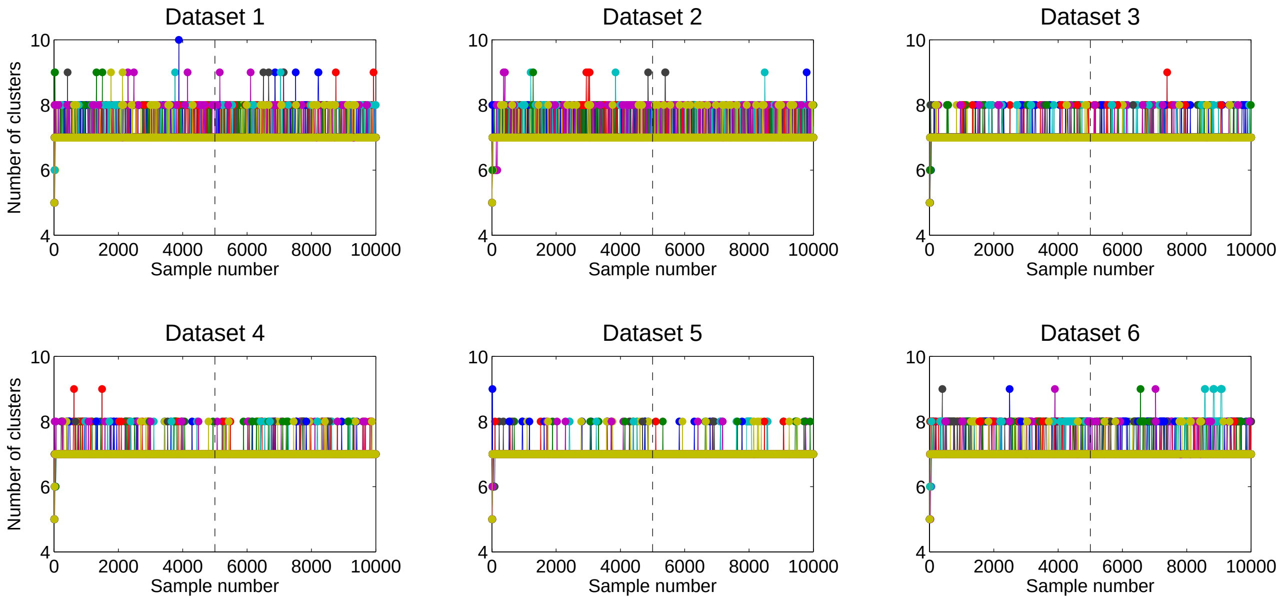

Fig. 3: MCMC diagnostic plots for the synthetic dataset example showing the number of clusters at each sample. Each coloured line corresponds to a different chain.

Expression+ChIP example

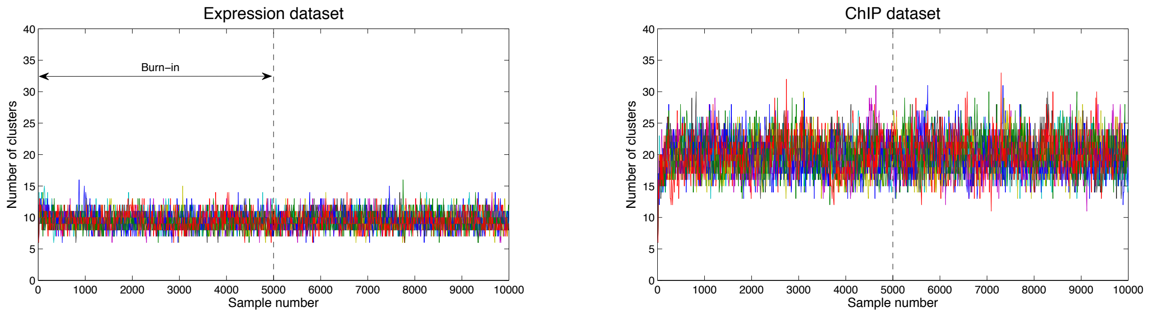

Fig. 4: MCMC diagnostic plots for the Expression+ChIP example showing the number of clusters at each sample. Each coloured line corresponds to a different chain.

Expression+ChIP+PPI example

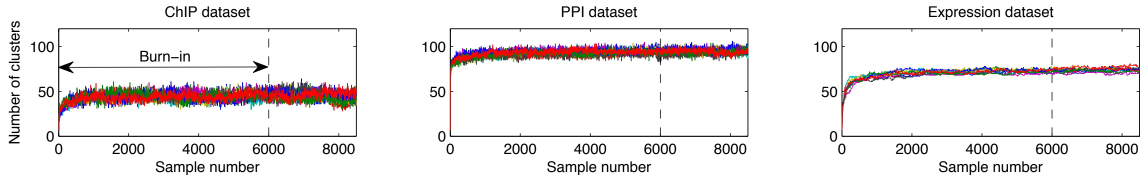

Fig. 5: MCMC diagnostic plots for theExpression+ChIP+PPI example showing the number of clusters at each sample. Each coloured line corresponds to a different chain.

D.4 Diagnostic plots II - Posterior similarity matrices

Posterior similarity matrices (PSMs; see Fritsch and Ickstadt 2009) are 2-dimensional arrays whose ij-entry is the posterior probability of gene i and gene j belonging to the same cluster. In this section we use these in order to provide a visual assessment of whether or not cluster memberships are consistent across chains.

For each chain separately, we used the samples obtained after the burn-in period in order to calculate the posterior probability of gene i and gene j belonging to the same cluster in dataset k (simply by calculating the proportion of the samples for which gene i and gene j were allocated to the same component). For each dataset, we thereby obtained a PSM corresponding to each chain. If we have reached convergence, the PSMs corresponding to different chains should be similar.

In Figures 6 and 7, we show the posterior similarity matrices associated with the cluster allocations of genes in the expression dataset of the "Expression+ChIP" example. Each heatmap corresponds to a different chain (as described by the figure titles). Genes are ordered along the rows and columns to reflect the clustering structure suggested by the posterior similarity matrix derived from Chain 1 (this is just to enable easier visual comparison of the heatmaps). Similarly, in Figures 8 and 9, we show the posterior similarity matrices associated with the cluster allocations of genes in the ChIP dataset of the "Expression+ChIP" example. We again order the genes along the rows and columns to reflect the clustering structure suggested by the posterior similarity matrix derived from Chain 1. From these heatmaps we can see that there is very good agreement among the chains regarding the allocation of genes to clusters for both datasets.

Figures 10 – 15 provide visualisations associated with the posterior similarity matrices obtained for the "Expression+ChIP+PPI" example. First, Figures 10 and 11 provide the posterior similarity matrices (PSMs) for the ChIP dataset; then Figures 12 and 13 show those for the PPI dataset; and finally Figures 14 and 15 provide the PSMs for the expression dataset. In all cases, the agreement between the PSMs across chains is again reasonable.

Expression+ChIP example: Posterior similarity matrices for the expression dataset

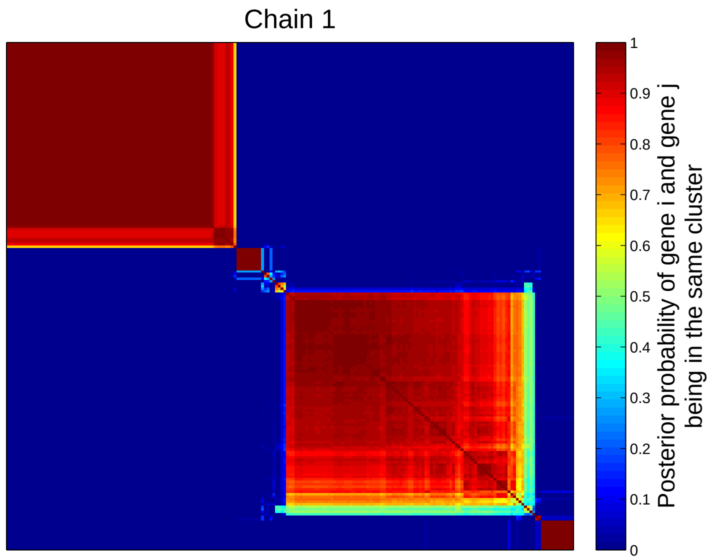

Fig. 6: Heatmap representation of the posterior similarity matrix (PSM) derived from Chain 1 in the Expression+ChIP example for the expression dataset. The ij-element of the matrix is the estimated posterior probability of genes i and j belonging to the same cluster. Rows and columns of the matrices are ordered to show the clustering structure (which aids comparison with the heatmaps in Figure 7).

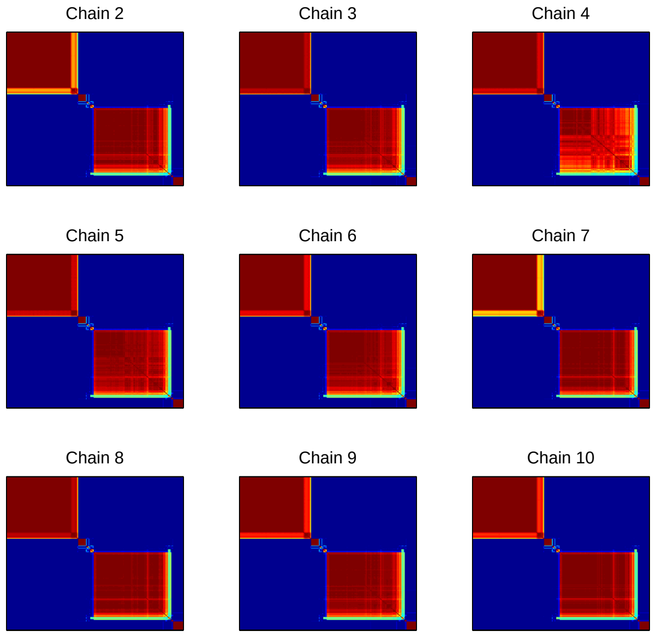

Fig. 7: PSMs derived from Chains 2 – 10 for the clusters in the expression dataset. Rows and columns have the same order as in Figure 6.

Expression+ChIP example: Posterior similarity matrices for the ChIP dataset

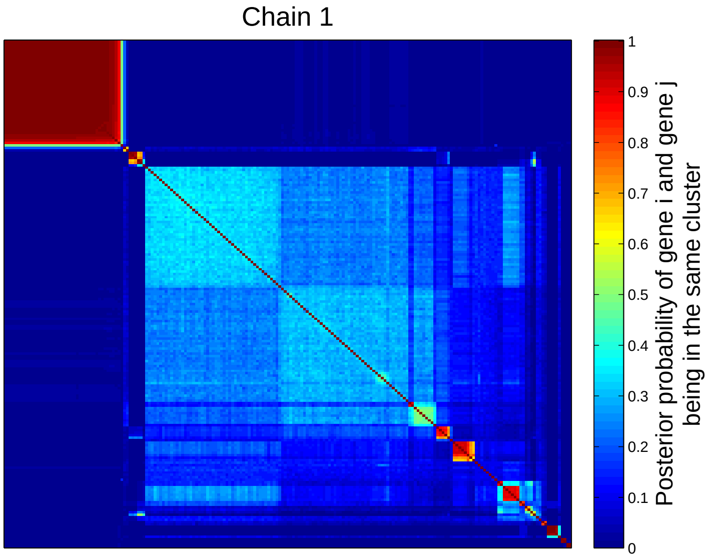

Fig. 8: Heatmap representation of the posterior similarity matrix (PSM) derived from Chain 1 in the Expression+ChIP example for the ChIP dataset. The ij-element of the matrix is the estimated posterior probability of genes i and j belonging to the same cluster. Rows and columns of the matrices are ordered to show the clustering structure (which aids comparison with the heatmaps in Figure 9).

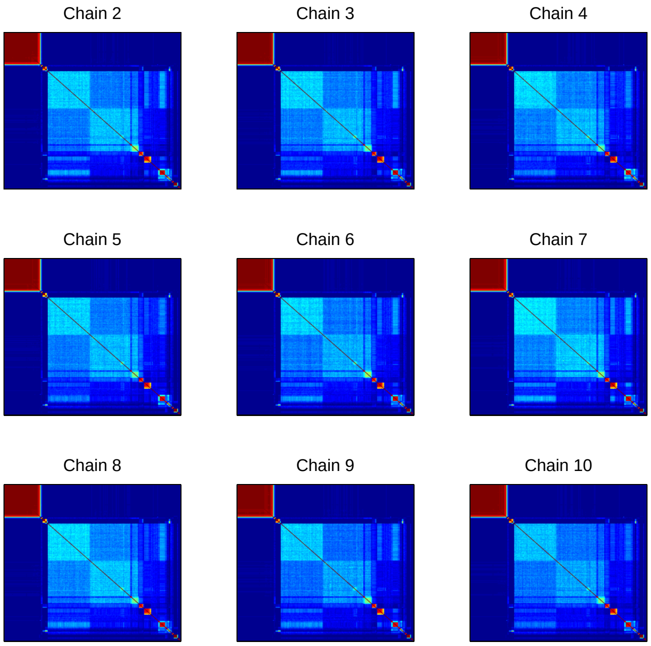

Fig. 9: PSMs derived from Chains 2 – 10 for the clusters in the ChIP dataset. Rows and columns have the same order as in Figure 8.

Expression+ChIP+PPI example: Posterior similarity matrices for the ChIP dataset

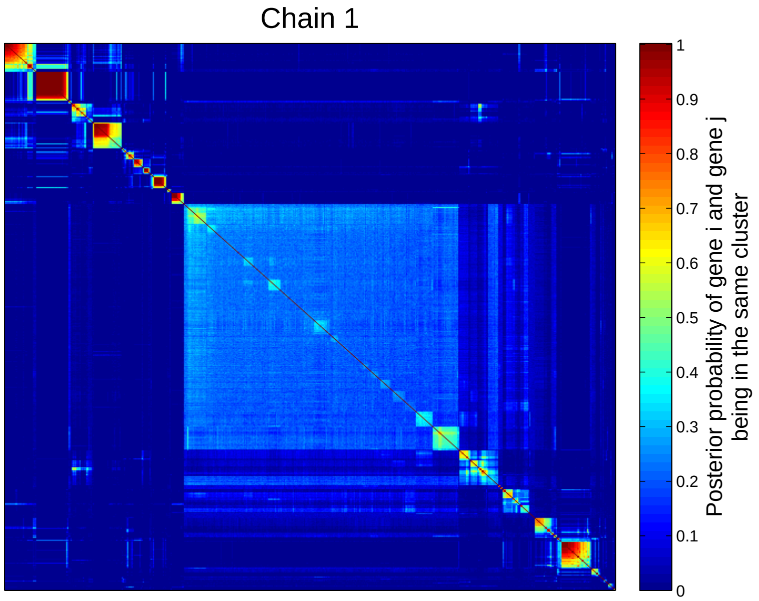

Fig. 10: Heatmap representation of the posterior similarity matrix (PSM) derived from Chain 1 in the Expression+ChIP+PPI example for the ChIP dataset. The ij-element of the matrix is the estimated posterior probability of genes i and j belonging to the same cluster. Rows and columns of the matrices are ordered to show the clustering structure.

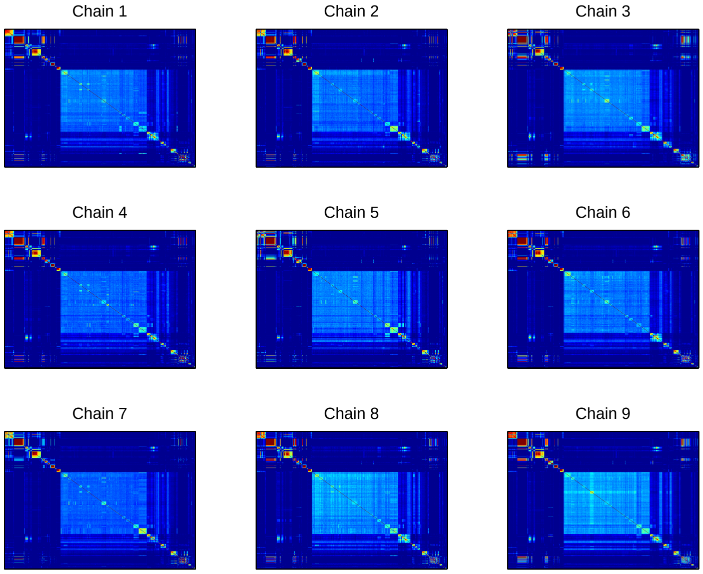

Fig. 11: PSMs derived from Chains 2 – 10 for the clusters in the ChIP dataset. Rows and columns have the same order as in Figure 10.

Expression+ChIP+PPI example: Posterior similarity matrices for the PPI dataset

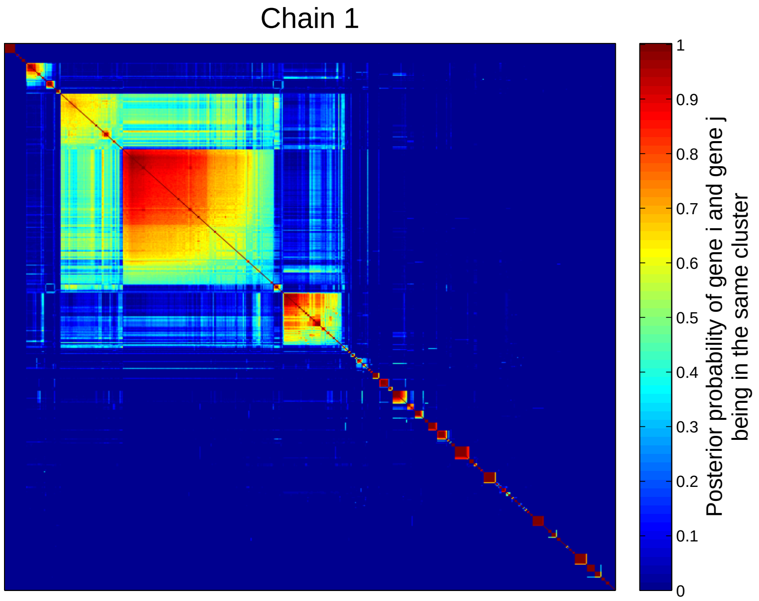

Fig. 12: Heatmap representation of the posterior similarity matrix (PSM) derived from Chain 1 in the Expression+ChIP+PPI example for the PPI dataset. The ij-element of the matrix is the estimated posterior probability of genes i and j belonging to the same cluster. Rows and columns of the matrices are ordered to show the clustering structure.

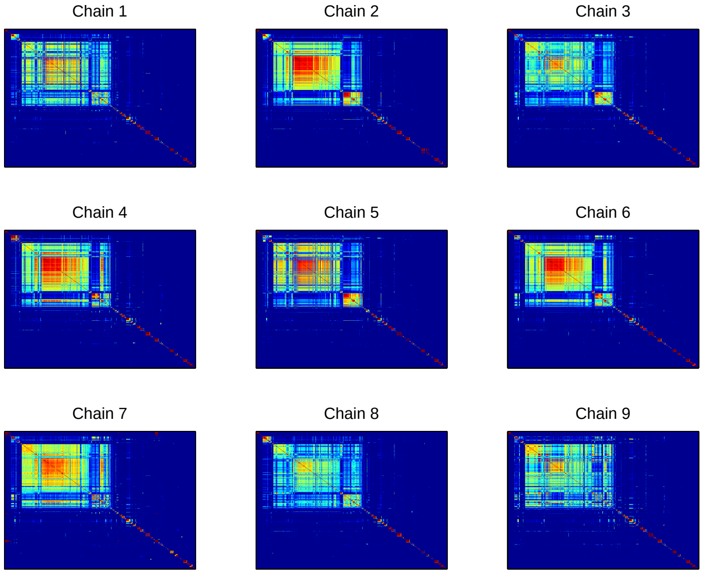

Fig. 13: PSMs derived from Chains 2 – 10 for the clusters in the PPI dataset. Rows and columns have the same order as in Figure 12.

Expression+ChIP+PPI example: Posterior similarity matrices for the expression dataset

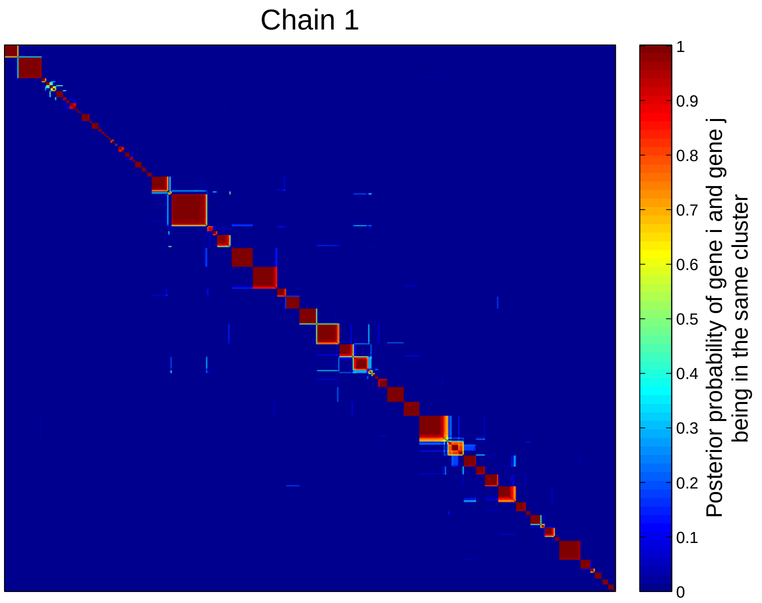

Fig. 14: Heatmap representation of the posterior similarity matrix (PSM) derived from Chain 1 in the Expression+ChIP+PPI example for the expression dataset. The ij-element of the matrix is the estimated posterior probability of genes i and j belonging to the same cluster. Rows and columns of the matrices are ordered to show the clustering structure.

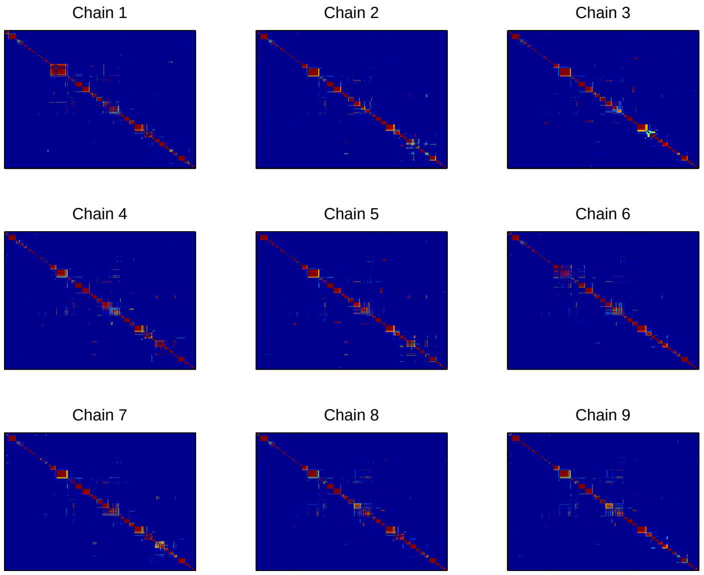

Fig. 15: PSMs derived from Chains 2 – 10 for the clusters in the expression dataset. Rows and columns have the same order as in Figure 14.

D.5 Scaling and run-times

Recall that K denotes the number of datasets, n is the number of genes, and N is the number of components in our mixture model (which places an upper bound on the number of clusters in the data). If n is large and K is modest, the rate determining step in the algorithm is the allocation of genes to components (i.e. sampling from the conditional for c_{ik}), which requires us to iterate over each gene in each dataset, and to calculate the probability of belonging to each component. In this case, the scaling of the algorithm will be $O(nNK)$. For larger K, the calculation of the normalising constant, Z, will dominate. If we were to calculate Z naively (by simply iterating through all possible c_{ik}'s – see Equation (2)), we would have a summation of N^K terms. However, note that (for example) in the $K = 3$ case we have,

$$(1 + \phi_{12})(1 + \phi_{13})(1 + \phi_{23}) = \sum_{(b_{12},b_{13},b_{23})\in\mathcal{B}} \phi_{12}^{b_{12}}\phi_{13}^{b_{13}}\phi_{23}^{b_{23}}, \tag{5}$$

where $\mathcal{B}$ is the set of all binary strings of length 3 (so, $|\mathcal{B}| = 2^3$). For brevity, in the general K case we rewrite the right hand side of Equation (5) as,

$$\sum_{\mathcal{B}} \prod_{k=1}^{K-1} \prod_{\ell=k+1}^{K} \phi_{k\ell}^{b_{k\ell}}.$$

It is then possible to rewrite Equation (2) as follows:

$$Z = \sum_{\mathcal{B}} \prod_{k=1}^{K-1} \prod_{\ell=k+1}^{K} \phi_{k\ell}^{b_{k\ell}} \sum_{j_1=1}^{N} \sum_{j_2=1}^{N} \cdots \sum_{j_K=1}^{N} \left(\prod_{k=1}^{K} \gamma_{j_k k} \prod_{k=1}^{K-1} \prod_{\ell=k+1}^{K} \mathbb{I}(j_k = j_\ell)^{b_{k\ell}} \right), \tag{6}$$

where we define $\mathbb{I}(j_k = j_\ell)^{b_{k\ell}} = 1$ whenever $b_{k\ell} = 0$ (even if $\mathbb{I}(j_k = j_\ell) = 0$). Although we still have the unpleasant multiple summation ($\sum_{j_1=1}^{N} \sum_{j_2=1}^{N} \cdots \sum_{j_K=1}^{N}\{\cdots\}$), the number of terms is massively reduced, thanks to the presence of the indicator function. To provide an example, consider $K = 3$. In this case, we have:

$$\begin{aligned}
Z =& \phi_{1,2}^0\phi_{1,3}^0\phi_{2,3}^0 \left(\sum_{j=1}^{N} \gamma_{j,1}\right)\left(\sum_{j=1}^{N} \gamma_{j,2}\right)\left(\sum_{j=1}^{N} \gamma_{j,3}\right) + \phi_{1,2}^0\phi_{1,3}^0\phi_{2,3}^1 \left(\sum_{j=1}^{N} \gamma_{j,1}\right)\left(\sum_{j=1}^{N} \gamma_{j,2}\gamma_{j,3}\right) \\
&+ \phi_{1,2}^0\phi_{1,3}^1\phi_{2,3}^0 \left(\sum_{j=1}^{N} \gamma_{j,2}\right)\left(\sum_{j=1}^{N} \gamma_{j,1}\gamma_{j,3}\right) + \phi_{1,2}^0\phi_{1,3}^1\phi_{2,3}^1 \left(\sum_{j=1}^{N} \gamma_{j,1}\gamma_{j,2}\gamma_{j,3}\right) \\
&+ \phi_{1,2}^1\phi_{1,3}^0\phi_{2,3}^0 \left(\sum_{j=1}^{N} \gamma_{j,3}\right)\left(\sum_{j=1}^{N} \gamma_{j,1}\gamma_{j,2}\right) + \phi_{1,2}^1\phi_{1,3}^0\phi_{2,3}^1 \left(\sum_{j=1}^{N} \gamma_{j,1}\gamma_{j,2}\gamma_{j,3}\right) \\
&+ \phi_{1,2}^1\phi_{1,3}^1\phi_{2,3}^0 \left(\sum_{j=1}^{N} \gamma_{j,1}\gamma_{j,2}\gamma_{j,3}\right) + \phi_{1,2}^1\phi_{1,3}^1\phi_{2,3}^1 \left(\sum_{j=1}^{N} \gamma_{j,1}\gamma_{j,2}\gamma_{j,3}\right).
\end{aligned}$$

Associated with each of the $\prod_{k=1}^{K-1} \prod_{\ell=k+1}^{K} \phi_{k\ell}^{b_{k\ell}}$ terms is a coefficient involving sums and products of γ's. The calculation of each of these coefficients is $O(NK)$, while the number of terms is equal to the number of binary strings of length m, where $m = K(K-1)/2$ is the number of $\phi_{k\ell}$'s. Calculation of the normalising constant therefore scales as $2^m NK$.

It follows that, for $n \approx 1{,}000$, the scaling of the algorithm will be $O(nNK)$ for $K \leq 5$ and $O(2^m NK)$ for larger K. It is important to note that, in practice, the wall clock time required by the algorithm is also affected by the choice of probability model assumed for the data. For example, examples employing Gaussian process models will generally be slower than examples employing simpler

bag-of-words models. For this reason, we also report the actual run-times for the examples considered in the main paper.

The method was implemented in Matlab and run on a 2.40GHz Intel Xeon CPU. The "Expression+ChIP" example (205 genes) took a little under 2 hours to run in the MDI (bag-of-words) case, and a little over 4 hours to run in the MDI (multinomial) case. The 6-dataset synthetic example (100 genes) generated approximately 500 samples per chain per hour. The "Expression+ChIP+PPI" example (551 genes) generated approximately 90 samples per chain per hour.

E GOTO scores

While the BHI is a useful means by which to assess the biological homogeneity of gene clusters, we found it to be both less sensitive and less informative than the GO Term Overlap (GOTO) similarity score of Mistry and Pavlidis (2008). If g_i and g_j are two distinct genes, then the GOTO score is calculated by first finding the set of all annotations, $annot_i$, for g_i (i.e. from the "leaves" of the hierarchy up to – but excluding – the root of the hierarchy), then doing the same for g_j, and finally calculating the Term Overlap, $GOTO(g_i, g_j) = |annot_i \cap annot_j|$. This similarity score is useful even if many of the genes have high-level GO terms in common, since a pair of genes that share lower-level, more specific GO terms will be scored more highly than a pair of genes that share high-level, less specific GO terms. Since we can calculate GOTO scores associated with each of the *biological process*, *molecular function*, and *cellular component* ontologies, we may therefore define GOTO (bp), GOTO (mf) and GOTO (cc).

The mean GOTO score associated with the (non-singleton) cluster $\mathcal{C}_q$ is calculated in the usual way, by taking the average of the GOTO scores associated with pairs of genes that appear in $\mathcal{C}_q$, i.e.

$$\overline{GOTO}(\mathcal{C}_q) = \frac{2}{n_q(n_q-1)} \sum_{g_i, g_j \in \mathcal{C}_q} GOTO(g_i, g_j), \tag{7}$$

where n_q is the number of genes in cluster $\mathcal{C}_q$, and hence there are $n_q(n_q-1)/2$ distinct pairwise comparisons.

In order to provide an overall summary for the clustering of the dataset, we may then calculate the weighted average,

$$\overline{GOTO}_{\text{overall}} = \sum_{q=1}^{Q} \left\{ \left(\frac{n_q}{n}\right) \overline{GOTO}(\mathcal{C}_q) \right\}, \tag{8}$$

where Q is the total number of non-singleton clusters and $n = \sum_{q=1}^{Q} n_q$.

E.1 GOTO scores for the Expression + ChIP example of Section 4.2

In addition to the BHI scores shown in Table 1 of the main paper, we also include GOTO scores for the example of Section 4.2 in the table below.

Table 1: GOTO scores for the Expression + ChIP example of Section 4.2.

	GOTO (bp)	GOTO (mf)	GOTO (cc)	Number of genes
Savage *et al.* (2010)	18.74	2.63	16.73	72
MDI (bag-of-words)	18.14	2.37	16.04	172
MDI (multinomial)	19.61	2.61	18.75	52

As in the BHI case, the GOTO scores show that the three methods are all performing similarly well. There is again evidence to suggest that the fused clusters identified by MDI (multinomial) have greater specificity (in the sense that the GOTO scores are generally higher), but lower sensitivity (since there are fewer fused genes) than the two bag-of-words methods.

F BHI scores for the Expression + ChIP + PPI example of Section 4.3

We found the BHI scores to be much less informative than the GO Term Overlap scores in the case of the Expression + ChIP + PPI example, largely as a result of the pre-filtering step that included only genes found to have periodic expression profiles over the cell cycle. Some high-level GO terms were particularly prevalent amongst the genes in our dataset (e.g. 48% of the genes were annotated with the "nucleus" GO term), which makes it quite likely that 2 genes will have at least one high-level GO term in common just by chance (regardless of how the genes are clustered). However, for completeness, we include the BHI scores for the Expression + ChIP + PPI example in the table below.

Table 2: BHI scores for combinations of expression, ChIP and PPI datasets.

	BHI (all)	BHI (bp)	BHI (mf)	BHI (cc)	Number of genes
ChIP only	0.50	0.12	0.23	0.32	551
PPI only	0.81	0.47	0.43	0.72	551
Expression only	0.57	0.19	0.21	0.43	551
ChIP+PPI	0.92	0.80	0.64	0.89	31
ChIP+Expression	0.81	0.45	0.39	0.76	48
PPI+Expression	0.90	0.62	0.53	0.89	32
ChIP+PPI+Expression	1.00	0.90	0.43	1.00	16

G Comparison of MDI to *iCluster* and simple clustering methods

G.1 6-dataset synthetic example

To illustrate the differences between MDI, *iCluster* and a simple clustering method, we briefly summarise the results of applying each of these methods to the synthetic data example of Section 3.1 of the paper.

A simple clustering method: k-means

Simple clustering methods such as k-means are unable to perform true integrative clustering of a collection datasets. We are instead forced either to cluster each of the datasets independently, or to concatenate the datasets to form a single data matrix. For the present example, it is most appropriate to cluster the datasets independently (since we know that the datasets were constructed in such a way that some genes switch between clusters in different datasets, so enforcing a single clustering structure would be undesirable). The results of applying Matlab's `kmeans` function (using the default squared Euclidean distance) to each of our 6 datasets independently are shown in Figure 16. In contrast to MDI, the number of clusters in each dataset is not determined automatically. Since we here know that the true number of clusters is 7, we set $k = 7$. We can see from Figure 16 that the clustering quality is reasonable. For datasets 1, 4 and 5, the clusterings are perfect, but for the other datasets the algorithm converges to a suboptimal local minimum. More importantly, however, is that there is no correspondence between the cluster labels for the different datasets, meaning that we would require a subsequent (possibly manual) processing step in order to "match up" the clusters between datasets. The algorithm moreover provides no way in which to detect "fused" genes (see Section 2.4 of the main paper) that cluster together across several of the datasets, and is unable to share information across datasets.

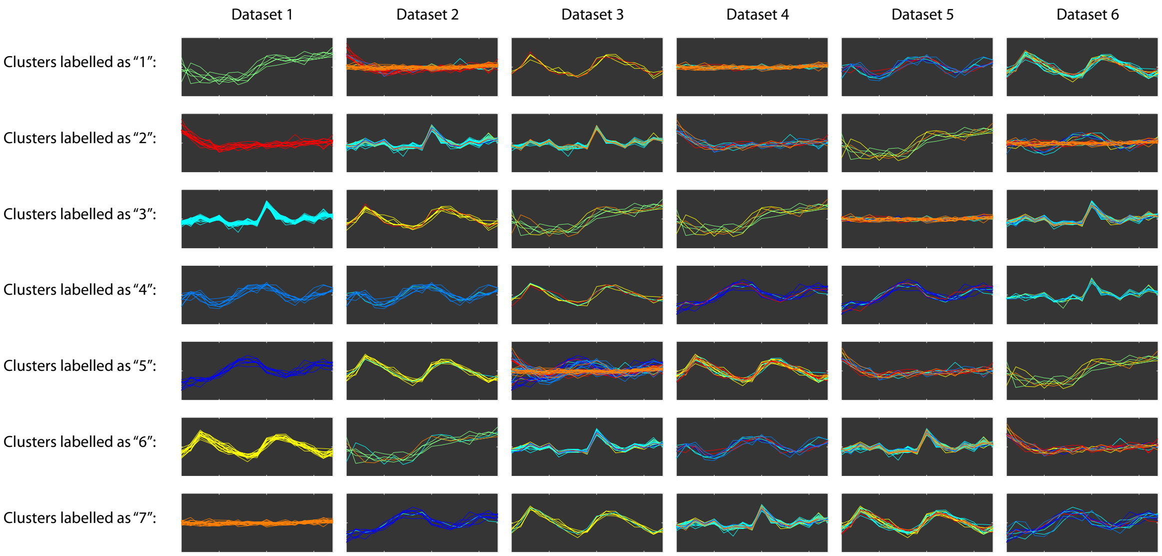

Fig. 16: Clusters obtained by (independent) k-means clustering of each of the synthetic datasets ($k = 7$). Genes are coloured as in Figure 2 of the main paper, so that the colours should appear coherent (i.e. should match up perfectly with the clusters) in dataset 1.

Integrative clustering using *iCluster* We next apply the integrative clustering (*iCluster*) method of Shen *et al.* (2009), which uses a joint latent variable model in order to perform clustering of collections of datasets. We use the `iCluster2` function in the `iCluster` package in `R`. Again, the number of clusters must be specified before running the function, so must either be known *a priori*, or else the function must be run multiple times for different values of k (and then an "optimal" k can be selected by choosing the one which minimises the authors' *proportion of deviation* (POD) score). Since we know that the true number of clusters in this case, we set $k = 7$. The results are shown in Figure 17 below. We can see that the clustering quality is quite poor, but that the algorithm successfully matches up clusters across the datasets. The reason for the poor clustering performance is due to *iCluster* seeking a common clustering structure for all datasets. This is inappropriate, since we know that some genes switch between clusters in different datasets. In order to compensate for this, we could take a larger value for k, which would allow *iCluster* to allocate problematic genes to singleton clusters. We therefore reran the algorithm for values of k between 2 and 70, and used the `compute.pod` function in the `iCluster` package in order to calculate POD scores for each. We found the minimal POD score to occur for $k = 60$ (see Figure 18). However, given that each dataset comprises only 100 genes, 60 clusters would seem undesirable.

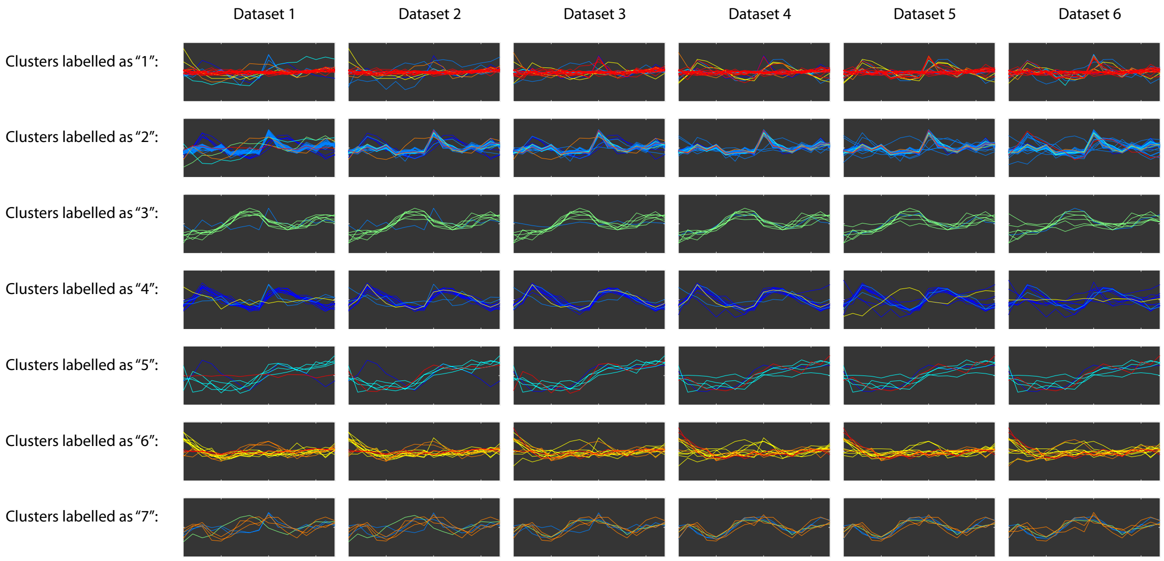

Fig. 17: Clusters obtained using *iCluster* with $k = 7$. The λ parameter required by the algorithm was set to 0.4, which was determined by considering a grid $\{0, 0.01, 0.05, 0.1, 0.2, 0.3, 0.4, 0.5\}$ of λ values, and selecting the value found to minimise the POD score.

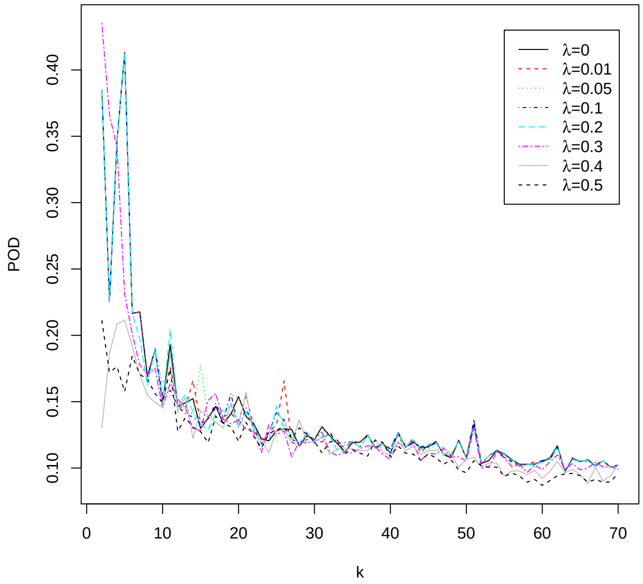

Fig. 18: POD scores for different values of k and λ.

MDI In Figure 19 below, we show the clusters obtained using MDI (we show results corresponding to a single representative sample from the posterior). MDI is able to infer the correct number of clusters for each dataset automatically, permits each dataset to have its own clustering structure, and automatically "matches up" corresponding clusters across datasets. As a result of this, MDI is able to identify the correct cluster allocations for all genes in all datasets.

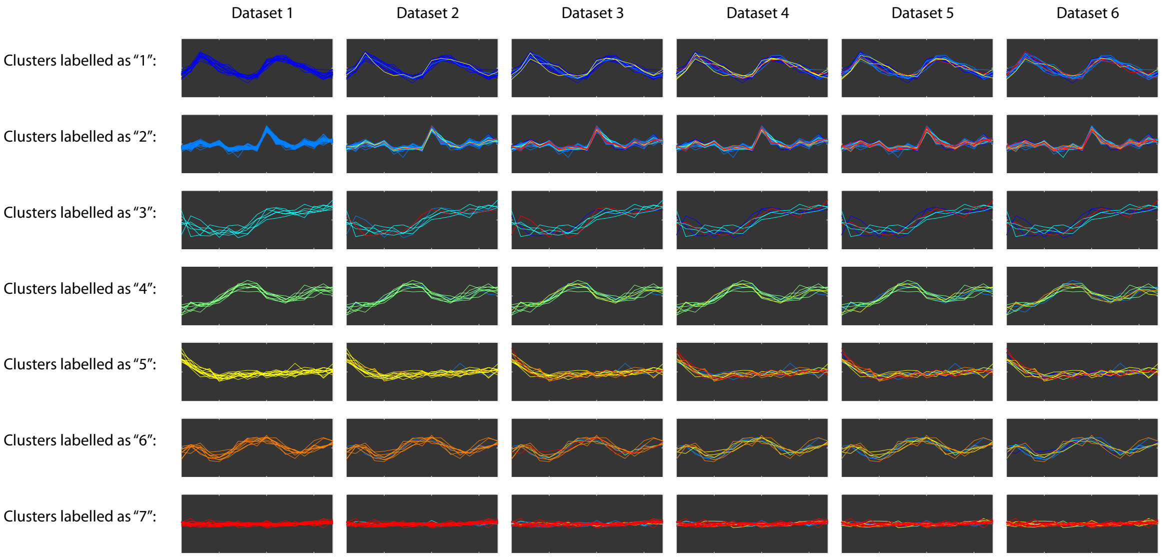

Fig. 19: Clusters obtained using MDI.

G.2 3-dataset example of Section 4.3

We applied *iCluster* to the collection of all 3 datasets and to all pairwise dataset combinations, and applied k-means clustering and 2 agglomerative hierarchical clustering approaches (single linkage and average linkage) to each of the datasets considered individually. For the simple clustering algorithms we set the number of clusters, k, to be equal to the number determined by MDI (namely: 74 for the expression dataset; 93 for the PPI dataset; and 42 for the ChIP dataset). We used squared Euclidean distances for the continuous data, and Hamming distances for the binary data. For *iCluster*, we tried both $k = 2$ (which minimised the POD score for each combination of datasets that we considered) and $k = 93$ (the number of clusters determined by MDI for the PPI dataset). In each case we determined the GOTO scores for the resulting clusters. Results are shown in Table 3, with the MDI results included for convenience.

The results show that – if k is chosen correctly – the *iCluster* method (which performs integrative clustering of all datasets) can yield better results than any of the simple clustering results. This is what we might hope: by sharing information across the datasets, we can find more biologically meaningful and specific clusters than if we consider each of the datasets independently. The results obtained by applying *iCluster* ($k = 93$) to all 3 datasets are worse than the results obtained using the MDI output to identify a clustering for the PPI dataset, but better than the results for the MDI clusterings of the ChIP and Expression datasets. Notably, however, the choice of k (the number of clusters) is very important: if we take $k = 2$, the *iCluster* results are comparable with the simple hierarchical clustering methods.

For all single datasets, MDI yields higher GOTO scores than the simple clustering methods, particularly for the PPI dataset. This seems to be because MDI is more robust to the noise in the PPI dataset, as a result of borrowing information from the other 2 datasets via the $\phi_{k\ell}$ parameters. The main benefit of MDI is that we are able to identify "fused" clusters, for which the gene-to-cluster allocations agree across all datasets. This enables us to identify increasingly specific gene groups, as reflected in the generally increasing GOTO scores.

Table 3: GOTO scores for the simple clustering methods (applied to each of the 3 datasets independently), *iCluster* (applied to the collection of all 3 datasets and to all pairwise dataset combinations), and MDI. Colours are used to group clusterings of the same datasets/dataset combinations. For the simple clustering approaches, the number of clusters, k, is set to be equal to the number in the MDI summary clustering. For *iCluster*, we tried both $k = 2$ (which minimised the POD score) and $k = 93$ (the number of clusters determined by MDI for the PPI dataset). For the simple clustering methods, we highlight in bold font the highest GOTO scores for each dataset.

	GOTO (bp)	GOTO (mf)	GOTO (cc)	Number of genes
**Simple methods:**				
ChIP (hclust, single linkage)	5.77	0.88	8.21	551
ChIP (hclust, average linkage)	5.73	**0.91**	8.23	551
ChIP (kmeans)	**6.24**	0.90	**8.38**	551
PPI (hclust, single linkage)	4.57	0.78	7.29	551
PPI (hclust, average linkage)	5.46	0.88	7.87	551
PPI (kmeans)	**7.37**	**1.02**	**8.82**	551
Expression (hclust, single linkage)	6.20	0.95	8.81	551
Expression (hclust, average linkage)	7.54	**1.13**	9.34	551
Expression (kmeans)	**7.59**	1.11	**9.43**	551
**iCluster:**				
ChIP+PPI (k = 2)	6.25	0.91	8.37	551
ChIP+PPI (k = 93)	8.00	1.07	9.48	551
ChIP+Expression (k = 2)	5.90	0.89	8.18	551
ChIP+Expression (k = 93)	7.14	1.06	9.00	551
PPI+Expression (k = 2)	5.87	0.89	8.16	551
PPI+Expression (k = 93)	8.80	1.33	9.74	551
ChIP+PPI+Expression (k = 2)	5.86	0.89	8.17	551
ChIP+PPI+Expression (k = 93)	9.05	1.31	10.00	551
**MDI:**				
ChIP	6.36	0.97	8.53	551
PPI	11.04	1.51	11.11	551
Expression	7.66	1.15	9.48	551
ChIP+PPI	27.04	3.47	18.99	31
ChIP+Expression	24.46	2.93	16.87	48
PPI+Expression	26.04	3.69	22.35	32
ChIP+PPI+Expression	34.81	2.46	26.70	16

H Further analyses for the Expression + ChIP + PPI example

In Section 4.3 of the main paper, we only presented results for one of the three possible pairwise comparisons (namely, genes fused across ChIP+PPI). In this section, we additionally consider the remaining pairwise comparisons (Expression+ChIP and Expression+PPI). We start in Section H.1 by providing the descriptions of the genes that were found to be fused across the ChIP and PPI datasets. We then provide summaries in Sections H.2 and H.3 of the clusters formed by the genes fused across the Expression+ChIP and Expression+PPI datasets (respectively). Where informative, we include figures illustrating the clusters.

H.1 ChIP + PPI

In Table 4 below, we list the genes that were found to be fused across the ChIP and PPI datasets, together with their cluster labels. Note that the cluster labels used here correspond to those used in Figure 4 in the main paper. In this case, the clusters correspond to groups of (putatively) co-regulated genes, whose protein products share common binding partners (which may be due to them being members of the same protein complex).

Table 4: Genes fused across ChIP and PPI datasets

Cluster	Name	Description
1	SCW11	Cell wall protein with similarity to glucanases; may play a role in conjugation during mating based on its regulation by Ste12p
1	ELO1	Elongase I, medium-chain acyl elongase, catalyzes carboxy-terminal elongation of unsaturated C12-C16 fatty acyl-CoAs to C16-C18 fatty acids
1	BUD9	Protein involved in bud-site selection
1	PRY2	Protein of unknown function
1	SVS1	Cell wall and vacuolar protein, required for wild-type resistance to vanadate
1	SCW10	Cell wall protein with similarity to glucanases
1	MSB2	Mucin family member involved in the Cdc42p- and MAP kinase-dependent filamentous growth signaling pathway; also functions as an osmosensor
1	WSC2	Sensor-transducer of the stress-activated PKC1-MPK1 pathway
1	TOS1	Covalently-bound cell wall protein of unknown function
1	BUD8	Protein involved in bud-site selection
1	SUT1	Transcription factor of the Zn[II]2Cys6 family involved in sterol uptake
2	NOB1	Involved in synthesis of 40S ribosomal subunits
2	ENP2	Required for biogenesis of the small ribosomal subunit
2	RPF2	Involved in the assembly of the 60S ribosomal subunit
2	IMP3	Component of the SSU processome
2	DBP9	Involved in biogenesis of the 60S ribosomal subunit
3	HHF2	Histone H4, core histone protein
3	HTB2	Histone H2B, core histone protein
3	HTA1	Histone H2A, core histone protein
3	HHT1	Histone H3, core histone protein
3	HTB1	Histone H2B, core histone protein
3	HHT2	Histone H3, core histone protein
3	HHF1	Histone H4, core histone protein
3	HTZ1	Histone variant H2AZ, exchanged for histone H2A in nucleosomes by the SWR1 complex
4	MCM3	Component of the Mcm2-7 hexameric complex
4	MCM5	Component of the hexameric MCM complex
5	SMC3	Subunit of the multiprotein cohesin complex
5	MCD1	Essential subunit of the cohesin complex
5	IRR1	Subunit of the cohesin complex
6	PCL2	Cyclin, involved in the regulation of polarised growth and morphogenesis and progression through the cell cycle
6	PCL1	Cyclin, involved in the regulation of polarised growth and morphogenesis and progression through the cell cycle

H.2 Expression + ChIP

In Figure 20 below, we provide an illustration of the clusters formed by the 48 genes fused across the Expression+ChIP datasets. In Table 5, we provide descriptions of the genes within each cluster. In this case, the clusters correspond to groups of genes that have correlated expression profiles due to co-regulation.

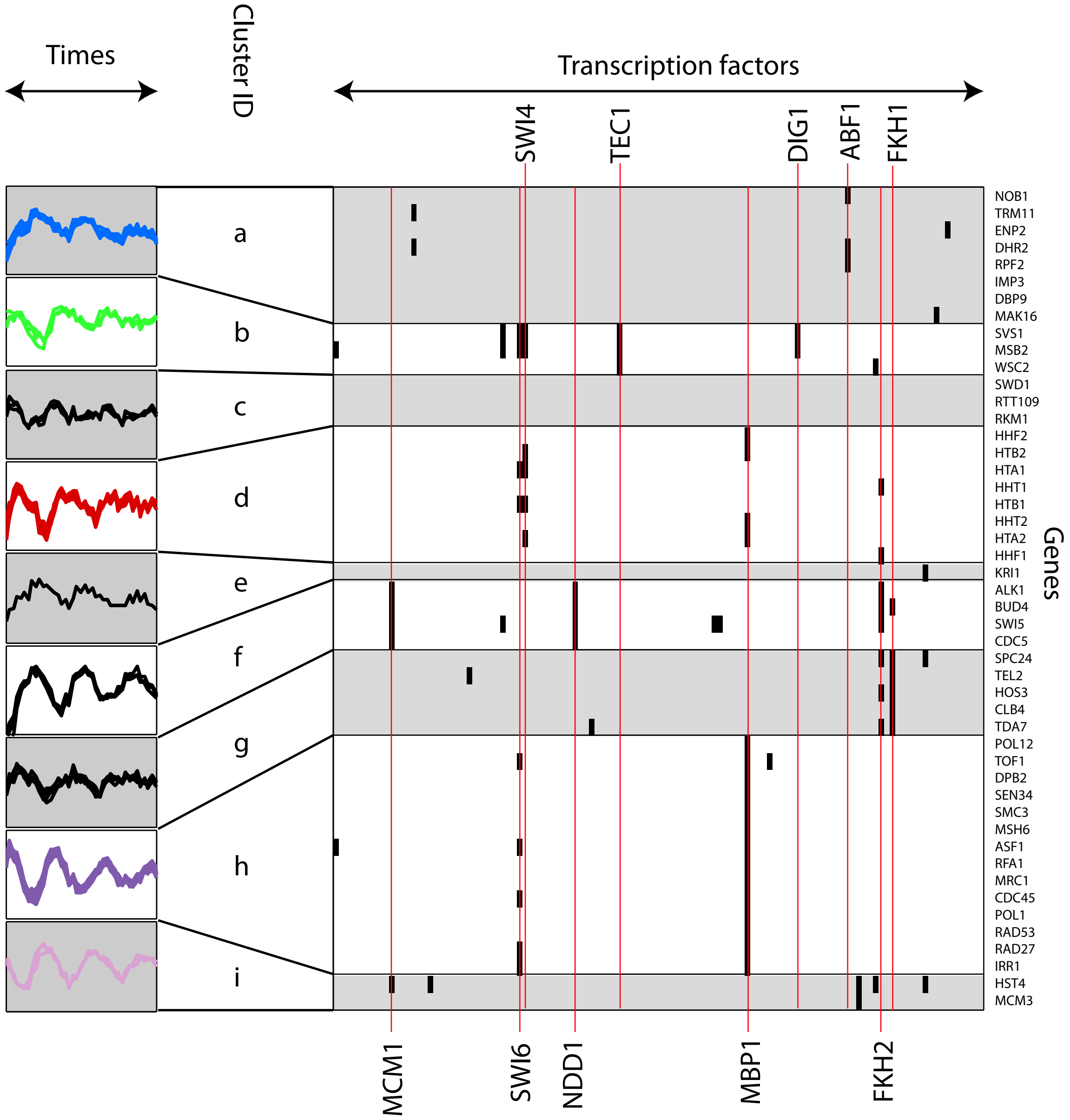

Fig. 20: Clusters formed by the genes fused across the expression and ChIP datasets. The time course data are shown on the left, with the ChIP data depicted on the right. We provide labels for some of the important transcription factors, with vertical red guidelines to improve readability.

Table 5: Genes fused across the Expression and ChIP datasets

Cluster	Gene	Brief description
a	NOB1	Involved in synthesis of 40S ribosomal subunits
a	TRM11	Catalytic subunit of an adoMet-dependent tRNA methyltransferase complex
a	ENP2	Required for biogenesis of the small ribosomal subunit
a	DHR2	Required for 18S rRNA synthesis
a	RPF2	Involved in the assembly of the 60S ribosomal subunit
a	IMP3	Component of the SSU processome
a	DBP9	Involved in biogenesis of the 60S ribosomal subunit
a	MAK16	Constituent of 66S pre-ribosomal particles
b	SVS1	Cell wall and vacuolar protein, required for wild-type resistance to vanadate
b	MSB2	Mucin family member involved in the Cdc42p- and MAP kinase-dependent filamentous growth signaling pathway; also functions as an osmosensor
b	WSC2	Partially redundant sensor-transducer of the stress-activated PKC1-MPK1 signaling pathway
c	SWD1	Subunit of the COMPASS (Set1C) complex, which methylates histone H3 on lysine 4
c	RTT109	Histone acetyltransferase; acetylates H3-K56 and H3-K9
c	RKM1	SET-domain lysine-N-methyltransferase
d	HHF2	Histone H4, core histone protein
d	HTB2	Histone H2B, core histone protein
d	HTA1	Histone H2A, core histone protein
d	HHT1	Histone H3, core histone protein
d	HTB1	Histone H2B, core histone protein
d	HHT2	Histone H3, core histone protein
d	HTA2	Histone H2A, core histone protein
d	HHF1	Histone H4, core histone protein
e	KRI1	Required for 40S ribosome biogenesis
f	ALK1	Protein kinase; accumulation and phosphorylation are periodic during the cell cycle
f	BUD4	Involved in bud-site selection; potential Cdc28p substrate
f	SWI5	Transcription factor that activates transcription of genes expressed at the M/G1 phase boundary and in G1 phase; appears to be regulated by phosphorylation by Cdc28p kinase
f	CDC5	Polo-like kinase with multiple functions in mitosis and cytokinesis; possible Cdc28p substrate
g	SPC24	Involved in chromosome segregation, spindle checkpoint activity and kinetochore clustering
g	TEL2	Required for telomere length regulation and telomere position effect
g	HOS3	Histone deacetylase (HDAC) with specificity in vitro for histones H3, H4, H2A, and H2B
g	CLB4	B-type cyclin involved in cell cycle progression; activates Cdc28p to promote the G2/M transition; may be involved in DNA replication and spindle assembly
g	TDA7	Cell cycle-regulated gene of unknown function
h	POL12	B subunit of DNA polymerase alpha-primase complex
h	TOF1	Subunit of a replication-pausing checkpoint complex (Tof1p-Mrc1p-Csm3p)
h	DPB2	Second largest subunit of DNA polymerase II (DNA polymerase epsilon)
h	SEN34	Subunit of the tRNA splicing endonuclease
h	SMC3	Subunit of the multiprotein cohesin complex
h	MSH6	Protein required for mismatch repair in mitosis and meiosis
h	ASF1	Nucleosome assembly factor
h	RFA1	Subunit of heterotrimeric Replication Protein A (RPA)
h	MRC1	S-phase checkpoint protein required for DNA replication
h	CDC45	DNA replication initiation factor
h	POL1	Required for the initiation of DNA replication during mitotic DNA synthesis and premeiotic DNA synthesis
h	RAD53	Protein kinase, required for cell-cycle arrest in response to DNA damage
h	RAD27	5' to 3' exonuclease, 5' flap endonuclease
h	IRR1	Subunit of the cohesin complex
i	HST4	Involved in silencing at telomeres, cell cycle progression, radiation resistance, genomic stability and short-chain fatty acid metabolism
i	MCM3	Component of the Mcm2-7 hexameric complex

H.3 Expression + PPI

In Table 6 below, we provide descriptions of the genes fused across the Expression+PPI datasets, and indicate the clustering obtained for these genes. In this case, the clusters correspond to groups of genes that have correlated expression profiles (which may or may not indicate co-regulation) and whose protein products share common binding partners (which may be due to them being members of the same protein complex).

Table 6: Genes fused across the Expression and PPI datasets

Cluster	Gene	Brief description
A	WSC2	Sensor-transducer of the stress-activated PKC1-MPK1 pathway
B	NOB1	Involved in synthesis of 40S ribosomal subunits
B	ENP2	Required for biogenesis of the small ribosomal subunit
B	RPF2	Involved in the assembly of the 60S ribosomal subunit
B	IMP3	Component of the SSU processome
B	KRI1	Required for 40S ribosome biogenesis
B	DBP9	Involved in biogenesis of the 60S ribosomal subunit
B	MAK16	Constituent of 66S pre-ribosomal particles
C	RKM1	SET-domain lysine-N-methyltransferase
D	PMT1	Involved in ER quality control
D	ERP2	Member of the p24 family involved in ER to Golgi transport
D	EMP24	Component of the p24 complex; binds to GPI anchor proteins and mediates their efficient transport from the ER to the Golgi; integral membrane protein that associates with endoplasmic reticulum-derived COPII-coated vesicles
E	HHF2	Histone H4, core histone protein
E	HTB2	Histone H2B, core histone protein
E	HTA1	Histone H2A, core histone protein
E	HHT1	Histone H3, core histone protein
E	HTB1	Histone H2B, core histone protein
E	HHT2	Histone H3, core histone protein
E	HHF1	Histone H4, core histone protein
F	MCM6	Component of the Mcm2-7 hexameric complex; forms a subcomplex with Mcm4p and Mcm7p
F	MCM2	Component of the Mcm2-7 hexameric complex
F	MCM3	Component of the Mcm2-7 hexameric complex
G	PDR3	Transcriptional activator of the pleiotropic drug resistance network, regulates expression of ATP-binding cassette (ABC) transporters through binding to cis-acting sites known as PDREs (PDR responsive elements); post-translationally up-regulated in cells lacking a functional mitochondrial genome
G	ALY1	Alpha arrestin that controls nutrient-mediated intracellular sorting of permease Gap1p; may regulate endocytosis of plasma membrane proteins by recruiting ubiquitin ligase Rsp5p to plasma membrane targets
G	YPT31	Involved in the exocytic pathway; mediates intra-Golgi traffic or the budding of post-Golgi vesicles from the trans-Golgi
G	ART5	Protein proposed to regulate the endocytosis of plasma membrane proteins
G	UBP13	Putative ubiquitin carboxyl-terminal hydrolase, ubiquitin-specific protease that cleaves ubiquitin-protein fusions
H	SMC3	Subunit of the cohesin complex
H	IRR1	Subunit of the cohesin complex
J	SPC110	Inner plaque spindle pole body (SPB) component
J	NUD1	Component of the spindle pole body outer plaque
J	SPC97	Component of the microtubule-nucleating Tub4p (gamma-tubulin) complex

I Effects of gene expression data normalisation

In Figure 4c of the main paper, we showed the expression profiles for the 31 genes identified as fused across the ChIP and PPI datasets. We can see that, despite being clustered together on the strength of the ChIP data and the PPI data, the genes in Cluster 1 (green) have very different expression profiles to one another. It is therefore unsurprising that this cluster is effectively removed when we consider genes that are fused across all 3 datasets. We also lose genes from Clusters 3, 4, 5 and 6. However, for these, the expression profiles of the genes that are lost are in some cases quite similar to those of the genes that remain in the cluster. For example, in Cluster 4, we can see that the expression profiles of MCM3 (pink) and MCM5 (grey) are actually quite similar. However, they are not clustered together on the strength of the expression data, since the two signals have different amplitudes. We investigate in this section whether or not this occurs as a result of the normalisation of the expression data.

I.1 Cluster 4

The genes in Cluster 4 are MCM3 and MCM5. In order to determine why MCM3 and MCM5 do not cluster together on the strength of their expression profiles, it is necessary to consider the expression profiles of the genes that *do* cluster together with each of them. In Figure 21a below, we show the expression profiles for the genes that are found to cluster together with MCM3 on the strength of the expression data only. Figure 21b shows the same, but for MCM5. Within these two clusters, the expression profiles are very similar to one another. Across clusters, we can see that the expression profiles shown in Figure 21b appear to be noisier than those in Figure 21a, and also have a greater amplitude. Recall that our Gaussian process models include hyperparameters that capture the noise and signal variance of the gene expression profiles within each cluster, and hence it is to be expected that these two clusters are distinct. The similarities and differences between the two clusters are perhaps clearer in Figure 21c, where we superimpose the two plots. However, it is possible that these apparent differences might be resolved with an additional or alternative normalisation of the data. One common additional normalisation step performed when considering time courses is to standardise the data, so that — for each gene — the standard deviation across time points is 1 and the mean is 0. We apply this normalisation to the genes in the two clusters, and then superimpose the resulting expression profiles in Figure 21d. We can see that the two clusters now coincide. This provides evidence to suggest that perhaps MCM3 and MCM5 should be clustered together on the strength of the expression as well as the ChIP and PPI data.

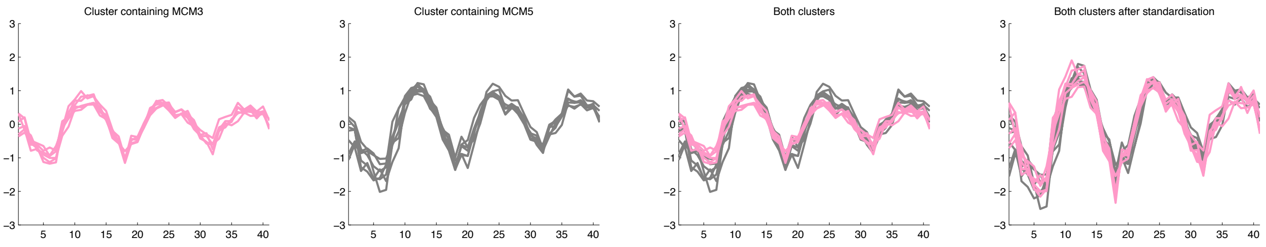

(a) (b) (c) (d)

Fig. 21: (a) Genes that cluster with MCM3 on the basis of the expression data only (including MCM3); (b) Genes that cluster with MCM5 on the basis of the expression data only (including MCM5); (c) A plot showing Figure 21a overlaid on Figure 21b; (d) Repeat of Figure 21c, after an additional normalisation step has been applied to the data.

I.2 Cluster 5

We perform a similar analysis for Cluster 5. This cluster contains SMC3 and IRR1 (purple) and also MCD1 (grey). SMC3 and IRR1 are found to cluster together across all 3 datasets, while MCD1 clusters together with the other two across the ChIP and PPI datasets, but not the expression dataset. Figure 22a shows the expression profiles for all of the genes that are clustered with SMC3 and IRR1 (on the strength of the expression data only), while Figure 22b shows the expression profiles for the genes clustered with MCD1. Figure 22c shows these two plots superimposed. Similar to the case for Cluster 4, we can see that the expression profiles in Figure 22b have greater amplitude than those in Figure 22a. Again, however, if we standardise the data as before, the two clusters coincide.

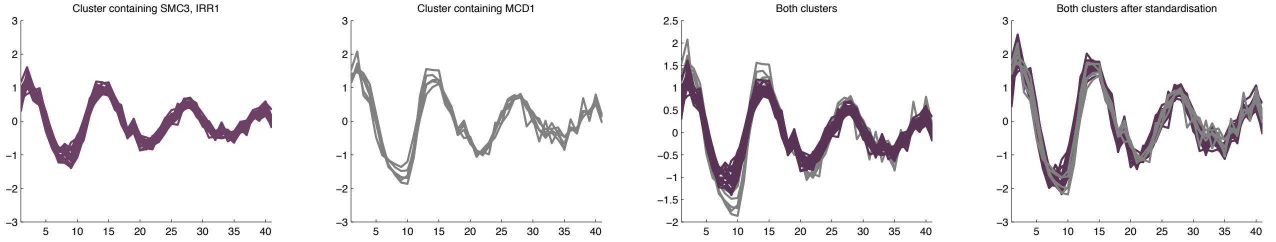

(a) (b) (c) (d)

Fig. 22: (a) Genes that cluster with SMC3 and IRR1 on the basis of the expression data only (including SMC3 and IRR1); (b) Genes that cluster with MCD1 on the basis of the expression data only (including MCD1); (c) A plot showing Figure 22a overlaid on Figure 22b; (d) Repeat of Figure 22c, after an additional normalisation step has been applied to the data.

I.3 Cluster 6

We again perform a similar analysis. Figure 23a shows the expression profiles for genes that cluster together with PCL1, while Figure 23b shows those for genes that cluster together with PCL2 (on the strength of the expression data only). Figure 23c shows the two plots superimposed, while Figure 23d shows the standardised expression profiles. We can see that, in this case, standardising the data does not cause the two clusters to align. There appears to be a lag between the expression of PCL2 and the expression of PCL1, which cannot be removed by a simple rescaling of the data.

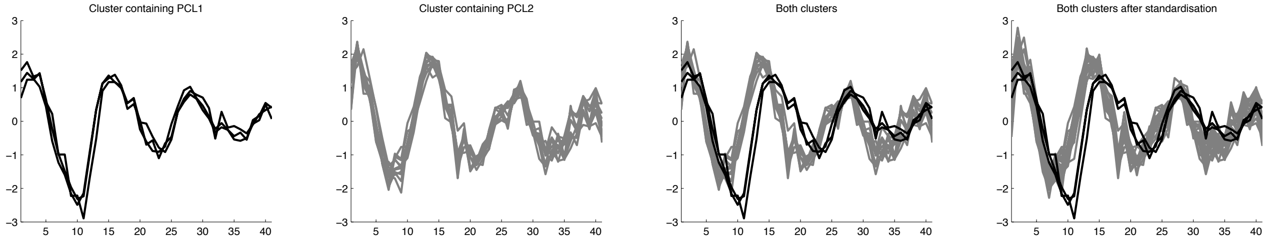

(a) (b) (c) (d)

Fig. 23: (a) Genes that cluster with PCL1 on the basis of the expression data only (including PCL1); (b) Genes that cluster with PCL2 on the basis of the expression data only (including PCL2); (c) A plot showing Figure 23a overlaid on Figure 23b; (d) Repeat of Figure 23c, after an additional normalisation step has been applied to the data.

J Extension to Section 4.2

In Sections 3.2 and 4.2, we considered a 205-gene example in which we integrated the galactose utilisation data of Ideker *et al.* (2001) with ChIP-chip data from Harbison*et al.* (2004). This example was chosen since it was also considered by Savage *et al.* (2010). However, since MDI permits more than 2 datasets to be integrated, we can extend this example by additionally including a protein-protein interaction dataset in our analysis. We use the same PPI dataset as considered in Sections 3.3 and 4.3. After restricting our analysis to genes for which all 3 datasets provided measurements, we were left with 199 genes. In this section, we provide the results of running MDI on this 3-dataset example, focusing solely on the clusters formed by the genes fused across all 3 datasets.

First, in Tables 7 and 8, we provide the BHI and GOTO scores for the fused clusters. We can see that the additional inclusion of the PPI dataset improves the BHI (bp) and BHI (mf) scores. The remaining BHI scores are maximal in both this analysis and the previous (Section 4.2) analysis. The GOTO scores provide a more detailed view of the results: while there is a modest increase in the average number of shared terms for genes in the same cluster in the case of the biological process and cellular component ontologies, there is a more pronounced increase in the case of the molecular function ontology.

Table 7: BHI scores for the clusters formed by the genes fused across all 3 datasets in the Galactose+Harbison+PPI comparison using MDI (multinomial). For comparison, we also include the results from the main paper for the Galactose+Harbison comparison.

	BHI (all)	BHI (bp)	BHI (mf)	BHI (cc)	Number of genes
Galactose+Harbison+PPI	1.00	1.00	0.85	1.00	42
MDI+Harbison	1.00	0.89	0.77	1.00	52

Table 8: GOTO scores for the clusters formed by the genes fused across all 3 datasets in the Galactose+Harbison+PPI comparison using MDI (multinomial). For comparison, we also include the results from Supplementary Section E.1 for the Galactose+Harbison comparison.

	GOTO (bp)	GOTO (mf)	GOTO (cc)	Number of genes
Galactose+Harbison+PPI	19.75	3.85	19.18	42
Galactose+Harbison	19.61	2.61	18.75	52

In Figure 24, we provide a representation of the clusters formed by the genes fused across all 3 datasets. Finally, in Table 9, we provide brief descriptions of the genes in each of the 5 clusters.

We can see from Table 9 that the genes in each cluster correspond to meaningful groups. Cluster I comprises key enzymes involved in glycolysis and gluconeogenesis; Cluster II is composed of genes coding for proteins that are components of the 40S and 60S ribosomal subunits; Cluster III corresponds to proteins involved in the formation of RNA polymerase II; Cluster IV contains the genes that encode the alpha and beta subunits of phosphofructokinase; and Cluster V comprises hexose transporter genes.

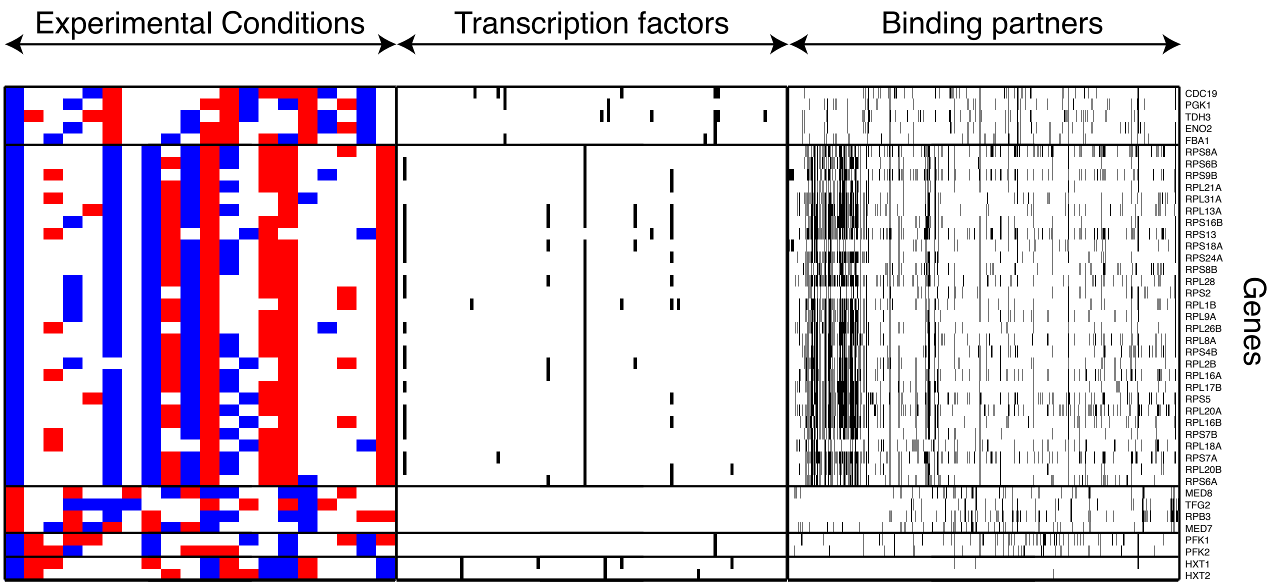

Fig. 24: Illustration of clusters fused across all three datasets in the Galactose+Harbison+PPI comparison. The datasets represented are (from left to right): the galactose utilisation (expression) dataset; the ChIP-chip dataset; and the PPI dataset. For the galactose utilisation dataset, the 3 colours (blue, white, red), correspond to the 3 discretised expression levels.

Table 9

Cluster	Name	Description
I	CDC19	Pyruvate kinase, functions as a homotetramer in glycolysis to convert phosphoenolpyruvate to pyruvate
I	PGK1	3-phosphoglycerate kinase; key enzyme in glycolysis and gluconeogenesis
I	TDH3	Glyceraldehyde-3-phosphate dehydrogenase, isozyme 3, involved in glycolysis and gluconeogenesis
I	ENO2	Enolase II, a phosphopyruvate hydratase that catalyzes the conversion of 2-phosphoglycerate to phosphoenolpyruvate during glycolysis and the reverse reaction during gluconeogenesis
I	FBA1	Fructose 1,6-bisphosphate aldolase, required for glycolysis and gluconeogenesis
II	RPS8A	Protein component of the small (40S) ribosomal subunit
II	RPS6B	Protein component of the small (40S) ribosomal subunit
II	RPS9B	Protein component of the small (40S) ribosomal subunit
II	RPL21A	Protein component of the large (60S) ribosomal subunit
II	RPL31A	Protein component of the large (60S) ribosomal subunit
II	RPL13A	Protein component of the large (60S) ribosomal subunit
II	RPS16B	Protein component of the small (40S) ribosomal subunit
II	RPS13	Protein component of the small (40S) ribosomal subunit
II	RPS18A	Protein component of the small (40S) ribosomal subunit
II	RPS24A	Protein component of the small (40S) ribosomal subunit
II	RPS8B	Protein component of the small (40S) ribosomal subunit
II	RPL28	Ribosomal protein of the large (60S) ribosomal subunit
II	RPS2	Protein component of the small (40S) subunit
II	RPL1B	N-terminally acetylated protein component of the large (60S) ribosomal subunit
II	RPL9A	Protein component of the large (60S) ribosomal subunit
II	RPL26B	Protein component of the large (60S) ribosomal subunit
II	RPL8A	Ribosomal protein L4 of the large (60S) ribosomal subunit
II	RPS4B	Protein component of the small (40S) ribosomal subunit
II	RPL2B	Protein component of the large (60S) ribosomal subunit
II	RPL16A	N-terminally acetylated protein component of the large (60S) ribosomal subunit
II	RPL17B	Protein component of the large (60S) ribosomal subunit
II	RPS5	Protein component of the small (40S) ribosomal subunit
II	RPL20A	Protein component of the large (60S) ribosomal subunit
II	RPL16B	N-terminally acetylated protein component of the large (60S) ribosomal subunit
II	RPS7B	Protein component of the small (40S) ribosomal subunit
II	RPL18A	Protein component of the large (60S) ribosomal subunit
II	RPS7A	Protein component of the small (40S) ribosomal subunit
II	RPL20B	Protein component of the large (60S) ribosomal subunit
II	RPS6A	Protein component of the small (40S) ribosomal subunit
III	MED8	Subunit of the RNA polymerase II mediator complex; associates with core polymerase subunits to form the RNA polymerase II holoenzyme
III	TFG2	TFIIF (Transcription Factor II) middle subunit; involved in both transcription initiation and elongation of RNA polymerase II
III	RPB3	RNA polymerase II third largest subunit B44, part of central core
III	MED7	Subunit of the RNA polymerase II mediator complex; associates with core polymerase subunits to form the RNA polymerase II holoenzyme
IV	PFK1	Alpha subunit of heterooctameric phosphofructokinase involved in glycolysis
IV	PFK2	Beta subunit of heterooctameric phosphofructokinase involved in glycolysis
V	HXT1	Low-affinity glucose transporter of the major facilitator superfamily
V	HXT2	High-affinity glucose transporter of the major facilitator superfamily

Bibliography

Cooke, E. J., Savage, R. S., Kirk, P. D., Darkins, R., and Wild, D. L. (2011). Bayesian hierarchical clustering for microarray time series data with replicates and outlier measurements. *BMC Bioinformatics*, **12**(1), 399.

Fritsch, A. and Ickstadt, K. (2009). Improved criteria for clustering based on the posterior similarity matrix. *Bayesian Anal*, **4**(2), 367–391.

Kirk, P. D. W. and Stumpf, M. P. H. (2009). Gaussian process regression bootstrapping: exploring the effects of uncertainty in time course data. *Bioinformatics*, **25**(10), 1300–6.

Mistry, M. and Pavlidis, P. (2008). Gene Ontology term overlap as a measure of gene functional similarity. *BMC Bioinformatics*, **9**, 327.

Nieto-Barajas, L., Prünster, I., and Walker, S. (2004). Normalized random measures driven by increasing additive processes. *Ann Stat*, **32**(6), 2343–2360.

Shen, R., Olshen, A. B., and Ladanyi, M. (2009). Integrative clustering of multiple genomic data types using a joint latent variable model with application to breast and lung cancer subtype analysis. *Bioinformatics*, **25**(22), 2906–12.
